# Postsynthetic On-Column 2′ Functionalization of RNA by Convenient Versatile Method

**DOI:** 10.3390/ijms21145127

**Published:** 2020-07-20

**Authors:** Olga A. Krasheninina, Veniamin S. Fishman, Alexander A. Lomzov, Alexey V. Ustinov, Alya G. Venyaminova

**Affiliations:** 1 Institute of Organic Chemistry and Center for Molecular Biosciences, University of Innsbruck, Innrain 80-82, Innsbruck 6020, Austria; 2 Institute of Cytology and Genetics SB RAS Lavrentiev Ave. 10, 630090 Novosibirsk, Russia; minja@bionet.nsc.ru; 3 Institute of Chemical Biology and Fundamental Medicine SB RAS Lavrentiev Ave. 8, 630090 Novosibirsk, Russia; lomzov@niboch.nsc.ru (A.A.L.); ven@niboch.nsc.ru (A.G.V.); 4 Shemyakin-Ovchinnikov Institute of Bioorganic Chemistry RAS Miklukho-Maklaya Str. 16/10, 117997 Moscow, Russia; austinov@yandex.ru

**Keywords:** RNA, chemical synthesis of RNA, TC-protecting group, 2′ hydroxyl, selective deprotection, solid-phase synthesis, postsynthetic modification

## Abstract

We report a universal straightforward strategy for the chemical synthesis of modified oligoribonucleotides containing functional groups of different structures at the 2′ position of ribose. The on-column synthetic concept is based on the incorporation of two types of commercial nucleotide phosphoramidites containing orthogonal 2′-*O*-protecting groups, namely 2′-*O*-thiomorpholine-carbothioate (TC, as “permanent”) and 2′-*O*-*tert*-butyl(dimethyl)silyl (*t*BDMS, as “temporary”), to RNA during solid-phase synthesis. Subsequently, the support-bound RNA undergoes selective deprotection and follows postsynthetic 2′ functionalization of the naked hydroxyl group. This convenient method to tailor RNA, utilizing the advantages of solid phase approaches, gives an opportunity to introduce site-specifically a wide range of linkers and functional groups. By this strategy, a series of RNAs containing diverse 2′ functionalities were synthesized and studied with respect to their physicochemical properties.

## 1. Introduction

Over the past decades, synthetic oligoribonucleotides have gained significant importance since the advent of a large number of functional noncoding RNAs (siRNAs, miRNAs, ribozymes, riboswiches, anti-replicative RNAs, and sgRNAs of the clustered regularly interspaced short palindromic repeats (CRISPR)/CRISPR-associated protein 9 (Cas9) system, etc.) which play key roles in various cellular processes and have potential as therapeutic agents. The development of novel advanced approaches for chemical synthesis of the functional RNAs and their conjugates is still of great interest (see, for instance, [1,2,3,4,5]). At the same time, there is a continued need for the development of new RNA-based tools for structural and functional analysis of biomolecules [6,7]. The 2′ position of a ribose moiety is an attractive conjugation site in nucleic acids (NAs), since 2′ modifications may increase nuclease resistance, binding affinity to NAs, and the ability to penetrate cells [8,9,10,11,12,13]. To date, a number of pre- and postsynthetic methods for the generation of 2′-modified RNAs have been reported. These methods have been used for chemical synthesis of various conjugates of siRNA [1,14,15,16], labeled pre-microRNAs [17,18,19], peptidyl-conjugates of RNA mimicking the tRNA^Ala^ acceptor arm [20], branched and lariat RNAs [21,22,23,24,25,26], and others. Recently, an original strategy of reversible acylation of 2′-OH groups of native RNA (RNA cloaking) for the controlled blocking of RNA hybridization, folding, and interactions with other biomolecules has been reported [27,28,29]. Interestingly, some of the 2′ modifications can also serve as protection, which is stable under conditions of the final deprotection and can be further selectively removed. For example, Beaucage and coauthors have proposed a convenient and efficient method for the preparation of reversible or permanent ribonucleoside 2′ conjugates using oxime formation from aldehydes or ketones [30,31]. They demonstrated that chimeric oligoribonucleotides modified with the 2′-*O*-(pyren-1-ylmethanimine-*N*-oxymethyl)uridine can be successfully converted to native RNAs by exposure to TBAF in DMSO. Debart and coauthors have introduced the oligoribonucleotides partially masked by base- or/and esterase-labile 2′-*O*-acetalester modifications (alkyl [32,33], lipophilic [12], and cationic [34]) as prodrug-like RNAs. The siRNAs with the 2′-*O*-acetalester modifications possess increased stability to nucleases, enhanced permeability into cells, and targeted suppression efficiency. Later, these authors reported an elegant postsynthetic solid-phase method for the synthesis of chimeric 2′-*O*-alkyl-, benzyldithiomethyl-modified (2′-*O*-RSSM, where R is a lipophilic moiety or polar groups), or doxorubicin-bearing oligoribonucleotides, which are turned into native RNAs under reduction conditions [13,35,36].

Although previously described methods have been successfully employed in the synthesis of 2′ conjugates of RNAs, most of them imply the multistep pre-synthesis of 2′-modified nucleoside phosphoramidites (U, A, C, and G). Here, we report a straightforward chemical strategy that involves the use of commonly used commercial reagents without any need for pre-synthesis of specially functionalized nucleotide phosphoramidites. Our approach is based on the combination of two types of the 2′-*O*-protecting groups, namely 2′-*O*-thiomorpholine-carbothioate (2′-*O*-TC, as permanent) [37] and 2′-*O*-*tert*-butyl(dimethyl)silyl (2′-*O*-*t*BDMS [38], as temporary), which can be removed under orthogonal conditions and are compatible with the standard protecting groups used for solid-phase phosphoramidite synthesis of oligoribonucleotides. After steps of selective partial deprotection, the following on-column 2′ functionalization of the oligoribonucleotide can be done using an efficient coupling reaction of the 2′ hydroxyl. This work demonstrates the utility of two alternative variants of on-column 2′ modification, including formation of 2′-phosphodiester linkage or 2′-*O*-carbamates. Subsequent post-modification may be performed on column or in solution by traditional cross-linking reactions. The approach includes the advantages of pre- and postsynthetic methods and provides a way for oligoribonucleotides to be site-specifically modified by various linkers and functional groups. Using this approach, a series of oligoribonucleotides (9 or 21 nt), containing several 2′ functionalities have been prepared. We confirmed high chemical stability of the 2′-phosphodiester linkage in the presence of a neighboring internucleotide (3′-5′-) phosphodiester. Our study includes the characterization of RNA duplexes of the 2′-modified oligoribonucleotides using UV-melting profile analysis and circular dichroism (CD) spectroscopy. Finally, the 2′-cholesterol-containing conjugates of siRNA synthesized by the described approach were evaluated for spontaneous cellular uptake using a HEK293 cell line.

## 2. Results and Discussion

**Figure 1:**
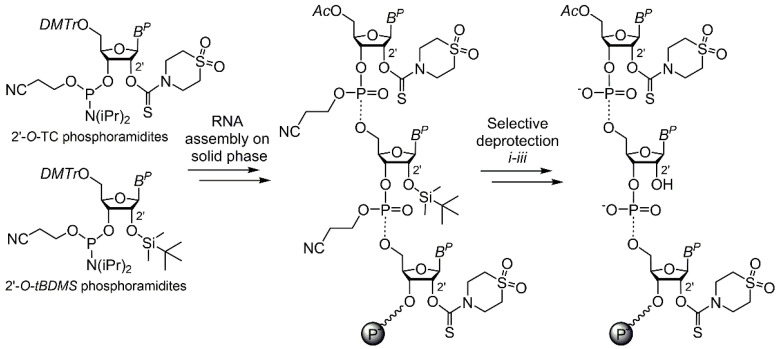
General synthetic strategy for the support-bound 2′-*OH*/2′-*O*-TC RNA. Selective deprotection: (i) *N,O*-bis(trimethylsilyl)acetamide (BSA), tetrahydrofuran (THF), 25 °C, 30 min; (ii) 2,3,4,6,7,8,9,10-octahydropyrimido(1,2-*a*)azepine (DBU), BSA, THF, 25 °C, 30 min; and (iii) Et_3_N∙3HF, triethylamine (TEA), *N*-methyl-2-pyrrolidinone, 65 °C, 1 h. B^P^—protected nucleobase (U, *N4*-acetyl C, *N6*-benzoyl A, *N2*-isobutyryl G), Ac—acetyl, DMTr—4,4′-dimethoxytrityl, *i*Pr—isopropyl, wavy line—the first nucleoside (deoxythymidine (dT) or deoxycytidine (dC)) attached to the polystyrene support via succinate linker (see Scheme S1).

The general concept of our strategy for 2′ functionalization of RNA involves the following major steps: (1) automated solid-phase synthesis, (2) selective deprotection of the 2′-hydroxyl group, (3) on*-*column 2′ functionalization, and (4) one-step final deprotection (and, if needed, a step of post-modification). First, the support-attached protected oligoribonucleotide containing ribonucleosides bearing two orthogonal 2′-*O*-protecting groups is assembled by phosphoramidite chemistry using 5′-*O*-DMTr nucleoside-modified polystyrene support. We assume that the proposed synthetic concept could, in general, tolerate several different combinations of the orthogonal 2′-*O*-protecting groups (permanent and temporary, respectively), for instance, (1) a pair of base-labile (e.g., 2′-*O*-thiomorpholine-carbothioate (TC) [37]) and fluoride-labile (2′-*O*-*tert*-butyldimethylsilyl (*t*BDMS) [38], 2′-*O*-triisopropylsilyloxymethyl (TOM) [39,40], or 2′-*O*-(2-cyanoethoxymethyl) (CEM) [41,42]) groups; (2) a pair of acid-labile (e.g., 2′-*O*-(1-(2-chloroethoxy)ethyl) (Cee) [43]) and one of the abovementioned fluoride-labile groups; or (3) a pair of base-labile (e.g., 2′-*O*-pivaloyloxymethyl (PivOM) [44,45]) and reducible (e.g., 2′-*O*-*tert*-butyldithiomethyl (DTM) [46]) groups. Here, the 2′-*O*-*t*BDMS is employed as a temporary protecting group since it is the most commonly used protection for the 2′-hydroxyl group; hence, the great variety of modified 2′-*O*-*t*BDMS phosphoramidites are widely available. At the same time, the 2′-*O*-TC plays a role of “permanent” protection, allowing to carry out the one-step final deprotection and cleavage of modified oligoribonucleotide from solid support, making chemical synthesis of RNA as simple as chemical synthesis of DNA [37]. After the synthesis, the 5′-*O*-DMTr group of the terminal nucleoside is removed with the following acetylation of 5′ hydroxyl by acetic anhydride (as a standard capping procedure) to avoid the unwanted side reactions of the 5′ terminal hydroxyl. Then, to mitigate a possible 2′- to 3′ isomerization of a phosphotriester group [47], the 2-cyanoethyl-protecting groups are selectively removed by β-elimination induced by a non-nucleophilic strong organic base (2,3,4,6,7,8,9,10-octahydropyrimido(1,2-a)azepine, DBU) in the presence of a silylation agent (*N,O*-bis(trimethylsilyl)acetamide, BSA) according to a described procedure [48]. After that, a removal of the temporary 2′-*O*-*t*BDMS group can be carried out by a reagent mixture containing the fluoride ion [49], leading to the support-bound 2′-*OH*/2′-*O*-TC RNA (Figure 1 and Scheme S1).

**Figure 2:**
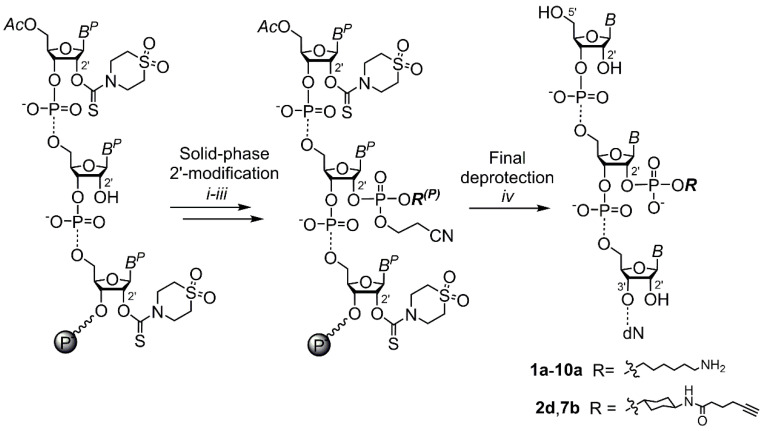
2′ functionalization of RNA via formation of a 2′-phosphodiester linkage. Solid-phase 2′ modification: (i) modifying (*R*) phosphoramidite, 5-(ethylthio)-1*H*-tetrazole, CH_3_CN abs., 10 min; (ii) I_2_, THF, pyridine, H_2_O; and (iii) CHCl_2_COOH, CH_2_Cl_2_. Final deprotection: (iv) NH_2_CH_2_CH_2_NH_2_ abs, 25 °C, 2 h. B^P^(B)—(un)protected nucleobase (U, C, A, and G). *R^P^*—protected functionalized linker (here, 6-(4,4′-dimethoxy-4″-methylsulfonyl-tritylamino)hexyl-, **1a**–**10a**). B^P^—protected nucleobase (U, *N4*-acetyl C, *N6*-benzoyl A, *N2*-isobutyryl G), Ac—acetyl, wavy line—the first nucleoside (deoxyribonucleoside (dN): dT or dC) attached to the solid support via succinate linker.

The following on-column postsynthetic 2′ functionalization of the support-bound 2′-*OH*/2′-*O*-TC RNA can be performed by efficient coupling reactions of the 2′ hydroxyl. Throughout this work, the functionalization was performed either via formation of 2′-phosphodiester linkage by a modifying phosphoramidite in a standard synthetic cycle (Figure 2 and Scheme S2) or alternatively via formation of 2′-*O*-carbamate derivatives (Scheme S3). We have chosen these two ways owing to their high yielding reactions and chemical stability of the resulting linkages under the conditions of final deprotection (neat ethylenediamine). A particular attention has been paid to the 2′ modification with the use of modifying phosphoramidite, since this is an attractive conjugation method to tailor RNA due to the simplicity of the procedure and a wide variety of commercially available reagents bearing various linkers or functional groups. Moreover, although pre-synthetic approaches to 2′-carbamate derivatives of oligonucleotides are already known [50,51,52,53,54], post-synthetic 2′ functionalization of RNA using 1,1′-carbonyldiimidazole coupling chemistry is, to our knowledge, not.

In general, the following post-modification could be carried out whether on column or, if final deprotection is detrimental for desired functional group, in solution by known conjugation reactions (for instance, conventional *N*-acylation reactions, copper(I)-catalyzed azide-alkyne cycloaddition (CuAAC) [55,56], copper-free strain-promoted azide-alkyne cycloaddition (SPAAC) [57], inverse-electron-demand Diels-Alder (IEDDA) cycloadditions [58,59], etc.). Apparently, the success of the conjugations depends on the chemoselectivity and yields of the reaction and on the size of a desired functional group.

First, since this synthetic strategy is based on the combination of 2′-*O*-TC and 2′-*O*-*t*BDMS-protecting groups, it was necessary to confirm the utility of the selected permanent protecting group in the strategy. Particularly, the stability of the 2′-*O-*TC group under the conditions of selective partial deprotection was verified. For this reason, the support-bound fully protected nonanucleotide 5′-dT_3_**U*_2′TC_***dT_5_ (where **U_2′TC_** is a 2′-*O*-TC protected uridine monomer) underwent steps of selective removal of 2-cyanoethyl protecting groups of internucleotidic phosphates and 2′-*O*-silyl protection (as shown in Figure 1), with the following release of the oligonucleotide from solid support by 10% DBU in THF. Acetylation of the 5′ hydroxyl of the oligonucleotide was skipped. RP-HPLC profiles of the crude products show that the 2′-*O*-TC group is stable under these conditions (Figure S1). Thus, we have demonstrated that the proposed combination of the 2′-*O*-protecting groups are compatible with the conditions of the selective deprotection.

**Figure 3:**
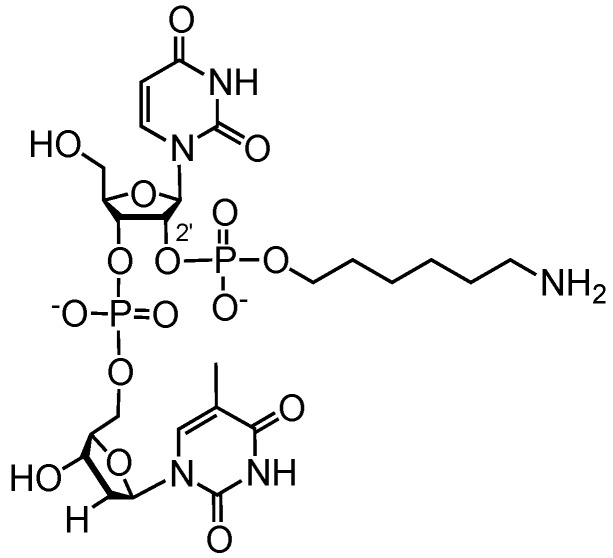
Chemical structure of dinucleotide **1a**.

With these encouraging results, we prepared a 2′-aminomodified dinucleotide (**1a**, 5′-U*dT-3′, where U* is a 2′-*O*-(6-aminohexyl)phosphate-containing uridine monomer, Figure 3) as a model compound to verify the 2′-functionalization procedure. Synthesis was straightforward by using the procedure described above, involving a modification with commercial 5′-amino-modifier C6 as a modifying phosphoramidite. The overall yield of **1a** after purification by TLC was 42%, and the structure was confirmed by NMR spectroscopy (see the Supplementary Materials, Sections 1.2–1.4, pages 4–6, Figures S3 and S4). Since the chemical stability of the phosphodiester linkages in RNA is a subject of special interest and introduction of a 2′ modification may affect it [60], we examined the stability of the dinucleotide **1a** under a number of varying conditions (pH, temperature, and incubation time; see Table S1) in the absence of divalent metal ions using RP-HPLC (Figures S5 and S6). It was shown that the dinucleotide **1a** remained intact under all conditions, exhibiting a high resistance to degradation. These observations are in agreement with previously published studies on the stability of 2′-branched oligonucleotides [61].

**Figure 4:**
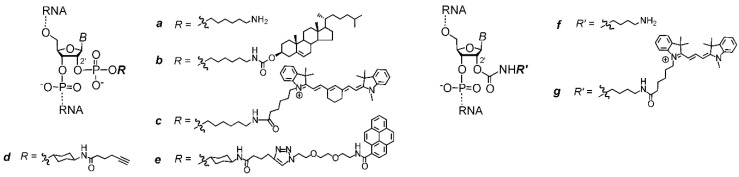
Chemical structure of 2′ modified nucleosides (N*).

Then, to assess the potential of the strategy, we prepared a series of model 2′-functionalized oligoribonucleotides bearing a new bioconjugation site (**2a**, **2d**, **2f**, **3a**–**6a**, and **7a**-**b**), a lipophilic moiety (**2b**), a fluorescent dye (**2c** and **2g**), or a polycyclic aromatic moiety (**2e**) (Table 1 and Figure 4). The oligoribonucleotides were assembled on a 0.2 μmol scale using commercially available 5′-*O*-DMTr nucleoside derivatized polystyrene support, 2′-*O*-*t*BDMS- and 2′-*O*-TC-protected RNA phosphoramidites, and 5-(3,5-bis(trifluoromethyl)phenyl)-1*H*-tetrazole as an activator, and coupling time was 150 s. After that, selective deprotection of 2′-*O*-*t*BDMS-groups was performed as described above (Figure 1). Then, on-column 2′ modification of the 2′-*OH*/2′-*O*-TC RNA was performed by a phosphoramidite (5′-amino-modifier C6 or alkyne phosphoramidite) in a standard synthetic cycle (**2a**, **2d**, **3a**–**6a**, and **7a**–**b**, Figure 2) or using 1,1′-carbonyldiimidazole-mediated amide coupling (**2f**, Scheme S3).

The post-modification by cross-linking reactions of the 2′-amino-modified oligoribonucleotide **2a** was performed either on solid support by treatment with cholesteryl chloroformate or in solution by reaction with *N*-hydroxysuccinimide (NHS) ester of cyanine 7 dye (giving **2b** and **2c**, respectively). The labeling of the oligoribonucleotide **2d** containing alkyne group was implemented in solution by a CuAAC reaction with pyrene azide, giving **2e**. The conjugation of 2′-*O*-(*N*-(4-aminobutyl)carbamoyl)-containing oligonucleotide **2f** was carried out in solution by a reaction with NHS-ester of cyanine 3 dye, giving **2g** ( Supplementary Materials, Section 1.7, page 7). The described procedures lead to crude oligoribonucleotides presented by major product (more than 80% according to RP-HPLC and PAGE; see, for instance, Figures S7 and S11). After purification by denaturing PAGE, the desired 2′-functionalized oligoribonucleotides (**2a**–**g**, **3a**–**6a**, and **7a**–**b**) were obtained in satisfactory yields (structures and characteristics of the conjugates are shown in Figure 4 and Table 1). The identities of the 2′ conjugates were verified by MALDI-TOF or ESI mass spectrometry analysis (see, for instance, Figure S10), RP-HPLC, and UV-spectra (for **2c**, **2e**, and **2g**; Figure S12). Full descriptions of the synthesis, 2′ functionalization, and characterization of the oligoribonucleotides are provided in Material and Methods and in the Supplementary Materials, Sections 1.6–2.2, pages 7–13.

Next, we investigated the influence of the 2′-modified ribonucleotide placed in a central position of oligoribonucleotides **3a**–**6a** on the thermal stability of their duplexes with the corresponding RNA targets (**t1**–**t4**) by temperature-dependent UV spectroscopy (Figure S13). The impact of the 2′-*O*-(6-aminohexyl)phosphate group at different ribonucleotides was identified by comparison of the thermostability of the complexes formed by the conjugates **3a**–**6a** and the reference unmodified oligonucleotides (**3**–**6**) with the complementary and centrally mismatched RNA targets (**t1**–**t4**). The T_m_ (ΔT_m_) values (Table 2) were calculated using the thermodynamic parameters ${\Delta G}_{298}^{o}$, ΔH°, and ΔS° (Table S3) that have been obtained by fitting procedure of UV-melting curves registered at two different wavelengths (260 and 270 nm) [62] according to two state models [63]. Oligoribonucleotides **3a**–**6a** having an additional negative charge of a 2′-*O*-(6-aminohexyl)phosphate-functionalized monomer display reduced affinity toward fully complementary RNA (ΔT_m_ values of (−6)–(−7) °C relative to unmodified duplexes). These results are consistent with our data obtained for duplexes of 2′-phosphate-modifed 2′-*O*-methyl oligoribonucleotides with their RNA targets [8]. Nevertheless, this destabilization does not prevent the oligoribonucleotides from forming stable complexes with RNAs under the experimental conditions (T_m_ values are 32.0–40.5 °C).

As expected, reference strands (**3**–**6**) display excellent discrimination of mismatched targets (**t1**–**t4**). In general, the 2′-modified oligoribonucleotides (**3a**–**6a**) demonstrate the same trends. We have observed an additional improvement in binding specificity (ΔT_m_ value of −3.0 °C) in case of a duplex **4a/t3** (with centrally positioned U*/G base pair). Interestingly, we noticed that the 2′ modification may also compensate a destabilizing effect of some mismatches, for instance, in the duplexes **3a/t2** (with centrally positioned A*/A base pair) and **5a/t1** (C*/U), the difference of T_m_ of modified duplexes relative to unmodified ones is +4.5 °C; this effect is even more pronounced in duplex **6a/t3** (G*/G) (ΔT_m_ value of +7.5 °C).

Circular dichroism (CD) spectra of the duplexes of the oligoribonucleotides (**3a**–**6a**) with the RNA targets (**t1**–**t4**) indicate the typical curve of an A-form helix geometry with a strong positive band at approximately 265 nm and a negative band at approximately 210 nm (Figure S14). Therefore, insertion of the 2′ modification via formation of a phosphodiester linkage does not induce significant distortions in the structures of the studied duplexes.

Concerning the introduction of 2′-*O*-carbamoyl modified nucleotides, this modification is known to decrease the thermostability of nucleic acids duplexes [50,51,52,53,54], most probably due to a steric clash and unfavorable electrostatics between the carbamoyl group and a nucleobase [64]. Interestingly, if a substituent with terminal cationic group is used, it may increase the duplex stability of the 2′-*O*-carbamoyl-modified oligonucleotides due to partial neutralization of the negatively charged internucleotide phosphates. The work of [54] reports that the 2′-*O*-(*N*-(4-aminobutyl)carbamoyl)uridine inserted in 2′-*O*-methyl oligoribonucleotides improves their hybridization affinity to RNA targets (compared to simplest carbamoyl substitute).

Poor cellular uptake of oligonucleotides having therapeutic potential is still a major problem to be solved. Conjugation of oligoribonucleotides to hydrophobic moieties and their analogs has been reported to enhance their cellular uptake (examples are numerous; see, for instance, [5,65,66,67,68,69]). A series of 2′-cholesterol-functionalized 21-mer oligonucleotides **8b**–**10b** (Table 1 and Table S2) corresponding to the sense strand of a model siRNA-targeting GFP mRNA [70] was synthesized as described above (Table 3 and Supplementary Materials, Section 1.9, page 9).

**Figure 5:**
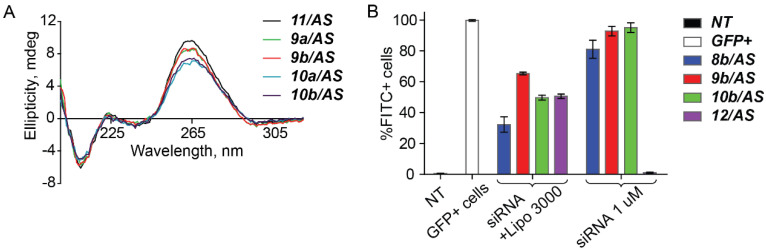
Modified siRNAs: (**A**) Overlay of the circular dichroism (CD) spectra of the modified siRNAs and reference unmodified siRNA, C_RNA_ = 15 μM. Sequences: 5′-r(GCCACAACGUCUAUAUCAU)dTdT-3′ (**11**), M_w_ calculated 6573.1, found (*m*/*z*) [M + H]^+^ 6574.4; 5′-r(AUGAUAUAGACGUUGUGGC)dTdT-3′ (***AS***), M_w_ calculated 6711.1, found (*m*/*z*) [M + H]^+^ 6714.9. (**B**) Flow cytometry data analysis: a bar graph showing the percentage of fluorescent (fluorescein channel) living cells observed after 4 h of transfection by fluorescein-labeled siRNAs (Table 3). Data are presented as medians obtained from 3 independent experiments.

Three dye-labeled siRNAs containing the 2′ modifications at three different sites, namely, at the third, seventh, or tenth nucleotides from the 3′ end of the sense strand of siRNA were annealed. The presence of a bulky functional group on an oligoribonucleotide backbone could lead to changes in double helix structure and, as a result, to loss of their biological functions. Analysis of the CD spectra of the duplexes showed that the overall conformation of an original A-form helix of RNA is retained in the 2′-cholesterol-functionalized siRNAs. The shapes of the spectra were similar to that of the unmodified duplex (Figure 5A).

The cellular uptake of these 2′-cholesterol-conjugated siRNA either in the absence (free uptake) or in the presence of a commercial transfection agent in the HEK293 Phoenix cell line was evaluated (see for details Supplementary Materials, Section 3.3, pages 16–18). The flow cytometry data shows that the efficiency of the cholesterol-conjugated siRNAs carrier-free uptake is rather high (more than 80%, at C_siRNA_ = 1 μM), while lipofectamine 3000-mediated delivery of all the siRNAs provided from approximately 30–65% of transfected cells (Figure 5B). As expected, in these conditions, the unmodified siRNA does not penetrate cells without a transfection agent. These observations were supported by localization of the fluorescently labeled siRNAs in HEK293 Phoenix cells by fluorescence microscopy (Figure S15). Importantly, neither changes of cell morphology nor increased levels of cell death were observed during the incubation.

## 3. Materials and Methods

### 3.1. General Methods

The protected 2′-*O*-*t*BDMS-ribo (A^Bz^, C^Ac^, G^iBu^, U) and 2′-deoxyribo (T) phosphoramidites as well as 6-(4,4′-dimethoxy-4″-methylsulfonyl-tritylamino)hexyl-(2-cyanoethyl)-(*N,N*-diisopropyl)-phosphoramidite (5′-amino-modifier C6) were purchased from Glen Research Inc (Sterling, VA, USA). The 2′-*O*-TC-ribo (A^Bz^, C^Ac^, G^iBu^, and U) phosphoramidites, 5-(3,5-bis(trifluoromethyl)phenyl)-1*H*-tetrazole (Activator 42), *N,O*-bis(trimethylsilyl)acetamide, 2,3,4,6,7,8,9,10-octahydropyrimido(1,2-*a*)azepine, 1,4-diaminobutane, *N,N*-diisopropylethylamine, and 1,1′-carbonyldiimidazole were purchased from Merck KGaA (Darmstadt, Germany). Nucleoside-derivatized (dT and dC) polystyrene supports (Custom Primer Support™, 80 μmol/g, PS 200) were purchased from GE Healthcare (Chicago, IL, USA). (1*R*,4*R*)-4-Hex-5-ylaminocyclohexyl-*N*,*N*-diisopropyl-(2-cyanoethyl)-phosphoramidite (5′-alkyne-modifier), NHS-esters of cyanine dyes Cy3 and Cy7, and *N*-(2-(2-(2-azidoethoxy)ethoxy)ethyl)pyrene-1-carboxamide (Pyrene azide 1) were purchased from Lumiprobe GmbH (Hannover, Germany). Solvents (tetrahydrofuran, dichloromethane, and acetonitrile (various vendors)) were dried by 3 Å molecular sieves or by distillation and storage over the CaH_2_.

^1^H, ^31^P NMR, and 2D ^1^H-^1^H COSY spectra were recorded in Shared Chemical Service Center of NIOC SB RAS on Bruker Avance 600 MHz spectrometer (Bruker Spectrospin, Karlsruhe, Germany). Chemical shifts (δ) are reported in ppm relative to residual solvent signals. The chemical shifts are referenced to the residual proton signal of the deuterated solvents: D_2_O (4.79 ppm) for ^1^H NMR spectra. For ^31^P NMR, 85% aq H_3_PO_4_ was used as an external standard. Signal multiplicities are given as s (singlet), d (doublet), t (triplet), m (multiplet), and br (broad).

The synthesis of all the oligoribonucleotides was carried out on an automatic ASM-800 synthesizer (Biosset, Russia) at 0.2 μmol scale with the use of nucleoside-modified polystyrene supports (GE Healthcare, Chicago, IL, USA; Custom Primer Support™, 80 μmol/g, PS 200) or CPG supports (Glen Research Inc, Sterling, VA, USA; CPG 500 Å, 30–40 μmol/g), 2′-*O*-TC-, 2′-*O*-*t*BDMS-protected RNA phosphoramidites, 5-(3,5-bis(trifluoromethyl)phenyl)-1*H*-tetrazole (Activator 42, coupling time was 2.5 min) or 5-(ethylthio)-1*H*-tetrazole (coupling time was 10 min) as an activator. Standard RNA synthesis cycle: 1) detritylation with dichloroacetic acid/dichloromethane (3/97) (120 s); 2) coupling with phosphoramidites in acetonitrile (0.1 M) and Activator 42 in acetonitrile (0.25 M) (180 s); 3) capping with a mixture of acetic anhydride with 2,6-lutidine in THF (Cap A: 10/10/80) and *N*-methylimidazole in THF (Cap B: 16/84) (2 × 15 s, Cap A/Cap B, 1:1); and 4) oxidation with 0.02 M iodine in pyridine/water/THF (1/9/90) (60 s).

Thin layer chromatography was carried out using DC-Alufolien Kieselgel 60 F254 TLC plates (Merck KGaA, Darmstadt, Germany) in appropriate solvent mixtures and was visualized by UV irradiation and stained by ninhydrin (0.1% in ethanol).

The identities of oligonucleotides were verified by MALDI-TOF or ESI mass spectrometry analysis. MALDI-TOF mass spectrometry analysis was performed with the use of 3-hydroxypicolinic acid as a matrix (recorded on a Bruker REFLEX III spectrometer, Bruker-Franzen Analytik GmbH, Bremen, Germany). ESI mass spectrometry analysis was performed on LC-ESI-IT (LC/MSD Trap XCT) or Agilent G1956B mass spectrometer (Agilent Technologies Inc., Santa Clara, CA, USA).

RP-HPLC analysis of the oligonucleotides and their conjugates was performed on an Alphachrome high performance liquid chromatograph (EcoNova, Novosibirsk, Russia) with the use of a ProntoSil-120-5-C18 AQ (75 × 2.0 mm, 5.0 μm) column, applying a gradient elution from 0 to 25% (10 min), from 25 to 47.5% (1.3 min), and from 47.5 to 50% (2 min) of acetonitrile in 0.02 M triethylammonium acetate buffer, pH 5.5 at a flow rate of 150 μL per min, and detection at 260 nm (oligonucleotide) and 345 nm (pyrene). The corresponding retention times for the oligonucleotides are presented in Table 1.

### 3.2. Oligoribonucleotide Synthesis and Selective Partial Deprotection

The synthesis of oligoribonucleotides was carried out using 5′-*O*-(4,4′-dimethoxytrityl)-protected nucleoside-derivatized polystyrene support and 2′-*O*-*t*BDMS- and 2′-*O*-TC-protected RNA phosphoramidites (5-(3,5-bis(trifluoromethyl)phenyl)-1*H*-tetrazole as an activator, coupling time was 2.5 min). The 5′-terminal dimethoxytrityl group of the oligoribonucleotides was removed and 5′ hydroxyl was acetylated (see Scheme S1). Support-bound protected oligoribonucleotides (I) were treated by a mixture of *N,O*-bis(trimethylsilyl)acetamide in tetrahydrofuran (50% vol., 500 μL) for 30 min at room temperature and 2,3,4,6,7,8,9,10-octahydropyrimido(1,2-*a*)azepine (5% vol.) in the mixture of *N,O*-bis(trimethylsilyl)acetamide in tetrahydrofuran (50% vol., 500 μL) for 30 min at room temperature (Scheme S1, steps a, b) [48]. After the treatment, the support-bound silylated oligonucleotide (II) was sequentially washed by tetrahydrofuran and acetone and air-dried. The selective removal of 2′-*O*-*t*BDMS (and simultaneous removal of trimethylsilyl) groups of support-bound oligonucleotide was done by a mixture of *N*-methyl-2-pyrrolidinone, triethylamine, and triethylamine·trihydrofluoride (6:3:4, 500 μL) at 65 °C for 1 h (Scheme S1, step c) [49]. Thereafter, support-bound 2′-*OH*/2′-*O*-TC oligoribonucleotide (III) was sequentially washed by tetrahydrofuran, acetonitrile, and acetone and air-dried.

### 3.3. Solid-Phase 2′ Modification by Modifying Phosphoramidite

2′ modification of support-bound 2′-*OH*/2′-*O*-TC oligonucleotide (III) was carried out as a standard cycle of automatic phosphoramidite synthesis with the use of 0.1 M modifying (5′-amino-modifier C6 or 5′-alkyne-modifier) phosphoramidite in anhydrous acetonitrile and 0.25 M 5-ethylthio-*1H*-tetrazole in anhydrous acetonitrile as an activator (coupling time was 10 min) (see Scheme S2, steps a–c).

### 3.4. Final Deprotection and Purification of Oligoribonucleotides

Cleavage from solid support and removal of the protecting groups of oligoribonucleotides were performed using neat ethylenediamine for 2 h at room temperature according to [37]. The 2′ modifications may increase solubility of deprotected oligoribonucleotides in ethylenediamine; for this reason, ethylenediamine solution was collected. The solid support was washed by acetonitrile (1.5 mL) and tetrahydrofuran (1.5 mL). Then, the crude oligoribonucleotide was washed away from the solid support by H_2_O (200 μL). After that, the crude 2′-modified oligoribonucleotides were precipitated from ethylenediamine and water solutions by the mixture of 2% NaClO_4_ in dry acetone and ethyl ether (1:1) and then washed by tetrahydrofuran (0.5 mL), ethanol (0.5 mL), and acetone (0.5 mL) to remove traces of ethylenediamine. The crude mixtures were purified by denaturing 15% PAGE, and the pure oligonucleotides were isolated as Na^+^ salts (see the characteristics of obtained conjugates in Table 1).

## 4. Conclusions

In this work, we have introduced a rather simple and cost-effective universal approach to the 2′ functionalization of oligoribonucleotides (1) that utilizes commercially available reagents without any need for pre-synthesis of all four (U, A, C, and G), specially functionalized nucleotide phosphoramidites, (2) that allows to modify any (potentially more than one) nucleotide of the RNA of interest during the solid phase phosphoramidite synthesis, and (3) that is based on a combination of modern synthetic methods of bioorganic chemistry characterized by high yields. We have shown the utility of two alternative variants of on-column 2′ modification, including formation of 2′-phosphodiester linkages and 2′-*O*-carbamates. To demonstrate the opportunities provided by the approach, we have prepared a series of oligoribonucleotides (9 or 21 nt) containing various 2′ functionalities (new bioconjugation sites, a lipophilic moiety, fluorescent dyes, or a polycyclic aromatic moiety). The chemical stability of the 2′-modified dinucleotide containing vicinal (2′- and 3′-) phosphodiester linkages was confirmed. Although, the introduction of additional phosphodiester (2′-) linkage leads to destabilization of the RNA duplexes (ΔT_m_ values of (−6)–(−7) °C), according to CD spectroscopy, their original A-form is retained. Finally, the model 2′-cholesterol-functionalized siRNA synthesized by the described approach was shown to efficiently penetrate HEK293 cells. Further design of nuclease resistant siRNAs, optimization of the 2′-functionalization strategies for synthesis of siRNAs bearing lipophilic ligands, and systematical studies of biological properties of these siRNAs are in progress. It is worth noting that the present approach is not limited by preparation of 2′ conjugates of RNA containing various reactive or functional groups attached through phosphodiester linkage and potentially can be extended to the generation of 2′,5′-branched RNAs (in preparation). Since our approach does not require specialized building blocks, we are confident that it can become a good alternative to previously reported methods for chemical synthesis of branched RNAs [21,22,23,24,25,26].

In summary, the reported convenient strategy for 2′ functionalization of RNA allows access to synthetic RNAs with site-specific tailored functionalities for applications in life sciences.

## Figures and Tables

**Table 1:** 2′ conjugates of oligoribonucleotides obtained in this study and their characteristics ^1^.

rON	Sequence ^1^	R	Isolated Yield/Conversion ^2^, %	RT ^3^, min	Molecular Weight	
					Found ^4^ *m/z*	Calcd *m/z*
**2**	5′-r(ACGUACGU)dT	-	12.5/85.1	6.3	2812.5	2813.8
**2a**	5′-r(ACGU*ACGU)dT	a	7.4/78.5	6.4	2991.9	2992.0
**2b**	5′-r(ACGU*ACGU)dT	b	3.1/~80 *	n/d	3405.6	3404.6
**2c**	5′-r(ACGU*ACGU)dT	c	5.6/84.5	9.9	3523.6	3523.7
**2d**	5′-r(ACGU*ACGU)dT	d	11.2/85.2	6.9	3084.9 ^†^	3084.1
**2e**	5′-r(ACGU*ACGU)dT	e	10.2/81.4	13.4	3529.5 ^†,5^	3486.5
**2f**	5′-r(ACGU*ACGU)dT	f	10.5/80.3	6.4	2926.2	2925.9
**2g**	5′-r(ACGU*ACGU)dT	g	5.4/90.9	12.8	3365.4	3367.6
**3a**	5′-r(GUGAA*AUG)dC	a	12.5/89.0	6.4	3041.7	3041.1
**4a**	5′-r(GUGAU*AUG)dC	a	18.4/84.6	6.4	3018.3	3018.1
**5a**	5′-r(GUGAC*AUG)dC	a	20.1/86.7	6.4	3017.9	3017.9
**6a**	5′-r(GUGAG*AUG)dC	a	19.5/87.4	6.5	3057.2	3057.9
**7a**	5′-r(GCCACAACGUCUAUAUCAU*)dTdT	a	5.6/78.9	7.2	6756.2 ^†^	6753.1
**7b**	5′-r(GCCACAACGUCUAUAUCAU*)dTdT	d	6.7/74.2	6.9	6847.8 ^†^	6844.1
**8a**	5′-Alk-r(GCCACAACGUCUAUAUCAU*)dTdT	a	6.0/69.3	7.3	7024.0 ^†^	7022.4
**8b**	5′-Fluo-r(GCCACAACGUCUAUAUCAU*)dTdT	b	3.6/~50 *	n/d	7894.3 ^†^	7894.5
**9a**	5′-Alk-r(GCCACAACGUCUAUA*UCAU)dTdT	a	5.7/76.7	7.3	7024.0 ^†^	7022.4
**9b**	5′-Fluo-r(GCCACAACGUCUAUA*UCAU)dTdT	b	3.5/~45 *	n/d	7895.0 ^†^	7894.5
**10a**	5′-Alk-r(GCCACAACGUCU*AUAUCAU)dTdT	a	5.7/75.6	7.3	7024.0 ^†^	7022.4
**10b**	5′-Fluo-r(GCCACAACGUCU*AUAUCAU)dTdT	b	3.9/~45 *	n/d	7895.5 ^†^	7894.5

^1^ 5′-r(NN…N)—oligoribonucleotide, dN—3′-terminal deoxynucleoside, N*—2′-modified nucleoside. ^2^ The percentage of the 2′-modified oligoribonucleotides was calculated based on the molar amount of the first nucleoside attached to a polymer after purification by denaturing PAGE/percentages of RNA of interest present in the crude samples after RNA deprotection determined by RP-HPLC (or by denaturing PAGE, marked with *; see, for instance, Figure S9). ^3^ RP-HPLC retention time of oligonucleotides conjugates. Conditions: see the general methods. ^4^ MALDI TOF or ESI (marked with ^†^) mass spectrometry. Alk—alkyne group, Fluo—fluorescein (6-isomer) (see Figure S8 and Table S2 for more details). ^5^ [M + 2Na^+^].

**Table 2:** Thermal denaturation temperatures (T_m_ values) of duplexes between **3**–**6** and **3a**–**6a** and complementary or centrally mismatched RNA targets.

ON	Sequence ^1^, 5′–3′	T_m_ (Δ T_m_^2^), °C			
**Target**	5′-r(GCAU***B***UCAC)	*B =* U	A	G	C
**3**	5′-r(GUGAAAUG)dC	39.0	23 (−18.5)	25.5 (−20.5)	27.0 (−18.5)
**4**	5′-r(GUGAUAUG)dC	26.0 (−13.0)	41.5	37.0 (−9.0)	26.0 (−19.0)
**5**	5′-r(GUGACAUG)dC	22.5 (−16.5)	25.5 (−16.0)	46.0	24.0 (−21.0)
**6**	5′-r(GUGAGAUG)dC	35.5 (−3.5)	25.0 (−16.5)	25.5 (−20.5)	47.5
**3a**	5′-r(GUGAA*AUG)dC	32.0	21.0 (−14.0)	18.0 (−22.0)	23.5 (−17.0)
**4a**	5′-r(GUGAU*AUG)dC	17.7 (−14.3)	35.0	28.0 (−12.0)	21.5 (−19.0)
**5a**	5′-r(GUGAC*AUG)dC	20.0 (−12.0)	20.0 (−15.0)	40.0	18.0 (−22.5)
**6a**	5′-r(GUGAG*AUG)dC	29.0 (−3.0)	21.0 (−14.0)	27.0 (−13.0)	40.5

^1^ 5′-r(NN…N)—oligoribonucleotide, dC—3′-terminal cytidine, N*—2′-*O*-(6-aminohexyl)phosphate nucleotide monomer (see Table 1 and Figure 4). RNA targets: 5′-r(GCAU***B***UCAC), where ***B*** = U (**t1**), ***B*** = A (**t2**), ***B*** = G (**t3**), and ***B*** = C (**t4**). ^2^ ΔT_m_ represents a difference between the T_m_ values of duplexes of 2′-modified (**3a**–**6a**) and reference unmodified (**3**–**6**) oligoribonucleotides with corresponding complementary RNA and the T_m_ values of the same oligonucleotides with centrally mismatched RNA targets. Thermal denaturation buffer: 0.1 M NaCl, 10 mM sodium cacodylate, pH 7.4, and 1 mM Na_2_EDTA; concentration of each strand was 1 μM.

**Table 3:** 2′-cholesterol-conjugated siRNA used in this study.

S/AS	Sequences	Representation
**8b/AS**	5′-Fluo-r(GCCACAACGUCUAUAUCAU*)dTdT3′-dTdT-r(CGGUGUUGCAGAUAUAGUA)	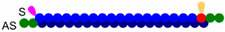
**9b/AS**	5′-Fluo-r(GCCACAACGUCUAUA*UCAU)dTdT 3′-dTdT-r(CGGUGUUGCAGAUAUAGUA)	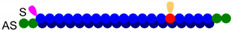
**10b/AS**	5′-Fluo-r(GCCACAACGUCU*AUAUCAU)dTdT 3′-dTdT-r(CGGUGUUGCAGAUAUAGUA)	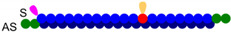
**12/AS**	5′-Fluo-r(GCCACAACGUCUAUAUCAU)dTdT 3′-dTdT-r(CGGUGUUGCAGAUAUAGUA)	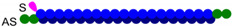

## Supplementary Materials

Supplementary materials can be found at https://www.mdpi.com/1422-0067/21/14/5127/s1.

## Abbreviations

RNA	Ribonucleic acid
siRNAs	Small interfering RNAs
miRNAs	MicroRNA
sgRNAs	Single guide RNA
CRISPR/Cas9	Clustered regularly interspaced short palindromic repeats/CRISPR-associated protein 9
MALDI-TOF	Matrix-assisted laser desorption/ionization
ESI	Electrospray ionization
PAGE	Polyacrylamide gel electrophoresis
RP-HPLC	Reversed phase high performance liquid chromatography
ON	Oligonucleotide
