# Supplementary material for: Postsynthetic On-Column 2′ Functionalization of RNA by Convenient Versatile Method
Source: Int J Mol Sci. 2020 Jul 20;21(14):5127. doi: 10.3390/ijms21145127 (PMC7404181; doi:10.3390/ijms21145127)
Supplement: Supplementary file 1 [file ijms-21-05127-s001.pdf]

International Journal of
Molecular Sciences

MDPI

Supplementary Materials

Postsynthetic On-Column 2'-Functionalization of RNA by Convenient Versatile Method

Olga A. Krasheninina [1,*], Veniamin S. Fishman [2], Alexander A. Lomzov [3], Alexey V. Ustinov [4] and Alya G. Venyaminova [3]

1 Institute of Organic Chemistry and Center for Molecular Biosciences, University of Innsbruck, Innrain 80-82, Innsbruck 6020, Austria
2 Institute of Cytology and Genetics SB RAS Lavrentiev Ave. 10, Novosibirsk 630090, Russia; minja@bionet.nsc.ru
3 Institute of Chemical Biology and Fundamental Medicine SB RAS Lavrentiev ave. 8, Novosibirsk 630090, Russia; lomzov@niboch.nsc.ru (A.A.L.); ven@niboch.nsc.ru (A.G.V.)
4 Shemyakin-Ovchinnikov Institute of Bioorganic Chemistry RAS Miklukho-Maklaya str. 16/10, Moscow 117997, Russia; austinov@yandex.ru
* Correspondence: okrasheninina@gmail.com or Olga.Krasheninina@uibk.ac.at

Table of contents

1. Synthesis of 2'-functionalized oligoribonucleotides 3

1.1. Stability of 2'-O-TC groups 3
1.2. Oligoribonucleotide synthesis and selective partial deprotection....3
1.3. Solid-phase 2'-modification by modifying phosphoramidite....4
1.4. Synthesis of 2'-modified dinucleotide 5'-$U_{2'pC6NH2}$pdT (**1a**) 4
1.5. Stability of 2'-modified dinucleotide **1a** containing vicinal (2'- and 3'-) phosphodiester bonds....6
1.6. On-column post-modification by cholesteryl chloroformate 7
1.7. Solid-phase 2'-modification by formation of carbamate derivative 7
1.8. Post-modification in solution....8
1.9. Synthesis of 2'-cholesterol-containing conjugates of sense strand of siRNA....9
1.10. Mass-spectrometry analysis of the 2'-conjugates of sense strand of siRNA....11
1.11. HPLC analysis of the 2'-functionalized oligoribonucleotides 12
2. Physicochemical studies 123
2.1. UV-visible absorption spectra 13
2.2. Thermal denaturation studies 13
2.3. CD spectra of duplexes of the 2'-functionalized oligoribonucleotides....16
3. Biological evaluation of 2'-cholesterol-containing siRNA conjugates 16
3.1. Cell culture....16
3.2. siRNAs....16
3.3. Cellular uptake of the 2′-cholesterol-conjugated siRNA in the HEK293 Phoenix cell line....16
3.3.1. Transfection....16
3.3.2. Flow cytometry....17
3.3.3. Confocal microscopy 17
References 19

1. Synthesis of 2'-Functionalized Oligoribonucleotides

1.1. Stability of 2′-O-TC Groups

Since the proposed scheme of selective deprotection implies the use of a combination of 2'-*O*-TC and 2'-*O*-TBDMS protecting groups, it was necessary to verify the utility of selected 2'-*O*-TC as a permanent protecting group in the strategy. The synthesis of oligodeoxynucleotides containing insertion of ribonucleoside (5'-$T_3U_{TC}T_5$) was performed with the use of dT phosphoramidite and 2'-*O*-TC-U phosphoramidite (5-ethylthio-*1H*-tetrazole as the activator, coupling time for 2'-*O*-TC-U was 10 min). Acetylation of the 5'-hydroxyl of the oligonucleotide was skipped. To test the stability of 2'-*O*-TC groups, the selective deprotection of support-bound fully protected oligonucleotide 5'-$T_3U_{2'TC}T_5$ (steps a–c, Scheme S1) was performed with the following final deprotection by treatment with 1,8-2,3,4,6,7,8,9,10-octahydropyrimido(1,2-a)azepine (10% vol.) in tetrahydrofuran for 1 h at 25 °C. RP-HPLC of the crude product show that the 2'-*O*-TC group is retained after all the performed steps of selective deprotection of the oligonucleotide (Figure S1). Thus, it was demonstrated that combination of the protecting groups is compatible with the optimized conditions of proposed stages of the strategy.

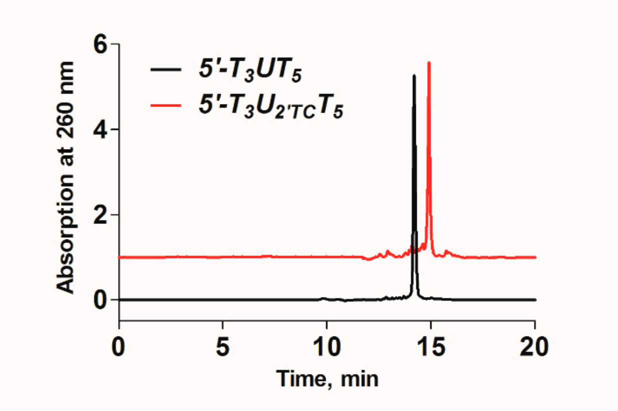

Figure S1. RP-HPLC profiles of crude 5'-T_3**U**$_{2'TC}T_5$ after treatment by reagents used at steps of the selective 2'-*O*-desilylation (Scheme S1, steps a–c) and reference 5'-T_3UT_5 (red and black lines, respectively): RP-HPLC was performed using a ProntoSil-120-5-C18 AQ (75 × 2.0 mm, 5.0 μm) column at a gradient elution from 0 to 50% (20 min) of acetonitrile in 0.02 M TEA acetate buffer, pH 5.5.

1.2. Oligoribonucleotide Synthesis and Selective Partial Deprotection

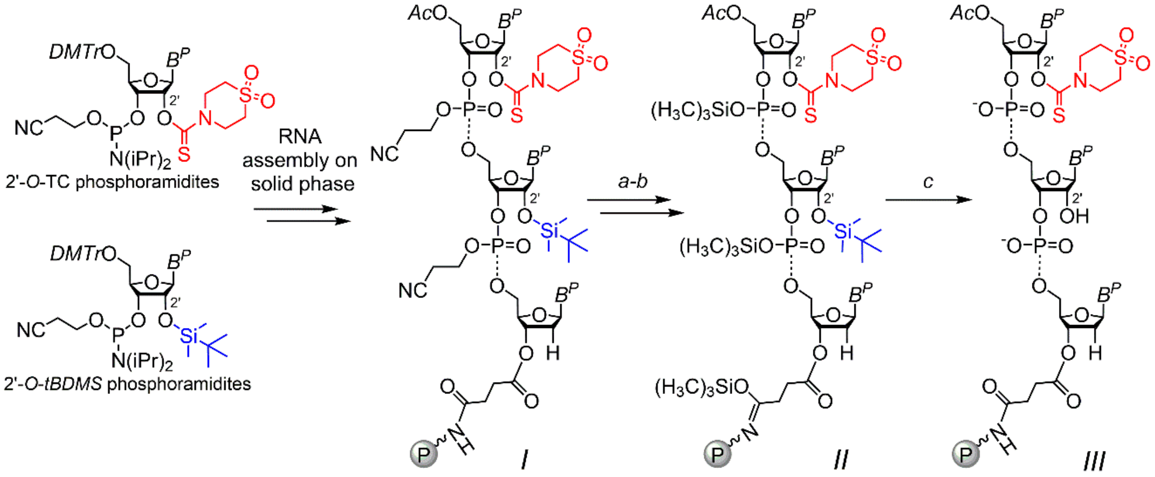

Scheme S1: Selective deprotection: (a) *N,O*-bis(trimethylsilyl)acetamide, THF, 25 °C, 30 min; (b) 2,3,4,6,7,8,9,10-octahydropyrimido(1,2-a)azepine, *N,O*-bis(trimethylsilyl)acetamide, THF, 25 °C, 30 min; and (**c**) $Et_3N{\cdot}3HF$, TEA, *N*-methyl-2-pyrrolidinone, 65 °C, 1 h. B^P—protected nucleobase (uracil, *N4*-acetyl cytosine, *N6*-benzoyl adenosine, *N2*-isobutyryl guanosine), Ac—acetyl, DMTr—4,4'-dimethoxytrityl, and *i*Pr—isopropyl.

1.3. Solid-Phase 2′ Modification by Modifying Phosphoramidite

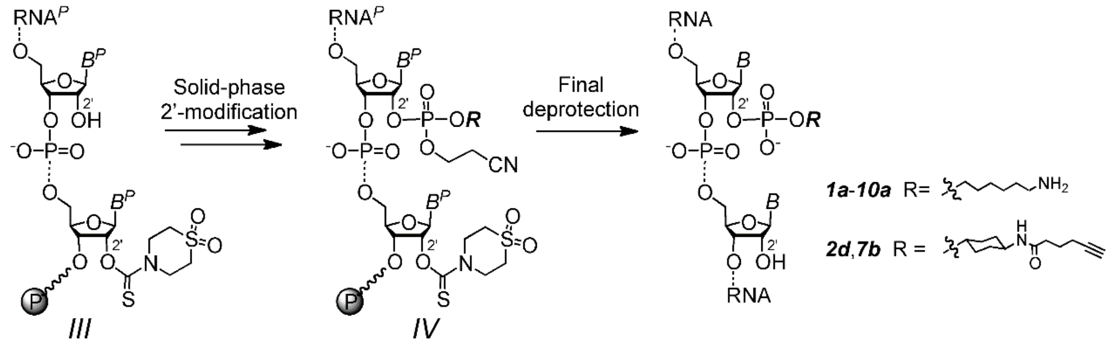

Scheme S2: Solid-phase 2′ modification: a) modifying (R) phosphoramidite, 5-(ethylthio)-1*H*-tetrazole, CH_3CN abs., 10 min; b) I_2, THF, pyridine, H_2O; and c) $CHCl_2COOH$, CH_2Cl_2. Final deprotection and cleavage from solid support: d) $NH_2CH_2CH_2NH_2$ abs, 25 °C, 2 h. B^P(B)—(un)protected nucleobase (U, C, A, and G).

*1.4. Synthesis of 2′-Modified Dinucleotide 5′-U$_{2'pC6NH2}$pdT (**1a**)*

Solid-phase synthesis of 5'-U$_{2'pC6NH2}$pdT (**1a**) (Figure S2) was performed as described above on a 3 µmol scale using 5'-O-DMTr thymidine-derivatized polystyrene support and 5'-amino-modifier C6 as a modifying phosphoramidite (Scheme S2). Deprotection of the amino group was carried out as a standard cycle of detritylation. The final deprotection and cleavage of the 2'-modified dinucleotide from solid support were performed in 30% NH_3 aq for 16 h at 25 °C. Isolation of **1a** from the crude mixture was performed by preparative TLC (2-propanol–H_2O–30% NH_3 aq 7:3:1 *v*/*v*/*v*) with the following elution (ethanol–H_2O–CH_3CN 1:1:1 *v*/*v*/*v*) and evaporation. R_f 0.5 (2-propanol–H_2O–30% NH_3 aq 7:3:1 *v*/*v*/*v*). Yield of ***1a*** was 42% (calculated based on molar amount of the first nucleoside attached to polymer). ^{1}H NMR (600 MHz, D_2O, δ, ppm) assignments are based on the ^{1}H-^{1}H Correlation Spectroscopy (COSY) NMR experiment: 7.87 (d, *J* = 8.10 Hz, 1H, H-C(6) U); 7.74 (d, *J* = 1.26 Hz, 1H, H-C(6) T); 6.32 (t, *J* = 6.74 Hz, 1H, H-C(1') T); 6.09 (d, *J* = 7.20 Hz, 1H, H-C(5) U); 5.91 (d, *J* = 8.13 Hz, 1H, H-C(1') U); 4.58 (m, 1H, H-C(3') T); 4.39 (m, 1H, H-C(2') U); 4.20-4.15, 4.09-4.13 (m, 3H, H-C(4′) T, (H1-),(H-2)-C(5') T); 3.68-3.81 (m, 5H, (H1-),(H-2)-C(5') U; H-C(4') U; -P(O)$_2$-O-C***H***$_2$-); 2.97 (t, *J* = 7.59 Hz, 2H, -C***H***$_2$-NH_3^+); 2.34-2.42 (m, 2H, H-C(2') T); 1.92 (d, *J* = 1.11 Hz, 3H, CH_3-C(5) T); 1.63 (m, 3H, -C*H*$_2$-CH_2-NH_3^+); 1.50 (m, 2H, -P(O)$_2$-O-CH_2-C*H*$_2$-), 1.32 (m, 5H) (-P(O)$_2$-O-CH_2-CH_2-C***H***$_2$-C***H***$_2$-). ^{31}P NMR (121 MHz, D_2O, δ, ppm): -0.615 (s, 1P, *2′*-); 0.187 (s, 1P, *3′*-), assignments are based on the work [1].

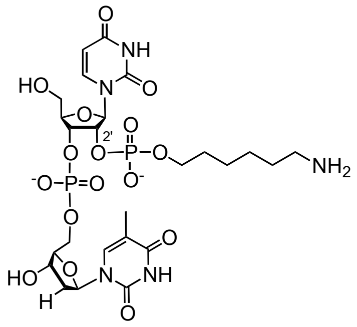

Figure S2. Chemical structure of dinucleotide **1a**.

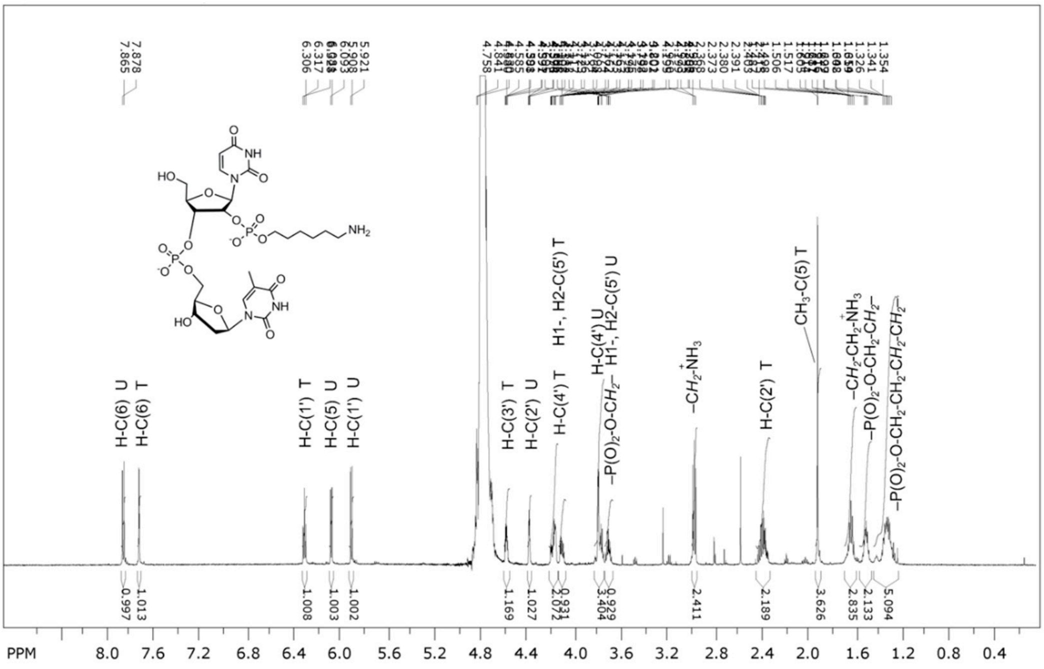

Figure S3. ^{1}H NMR (600 MHz, D_2O) of compound 1a.

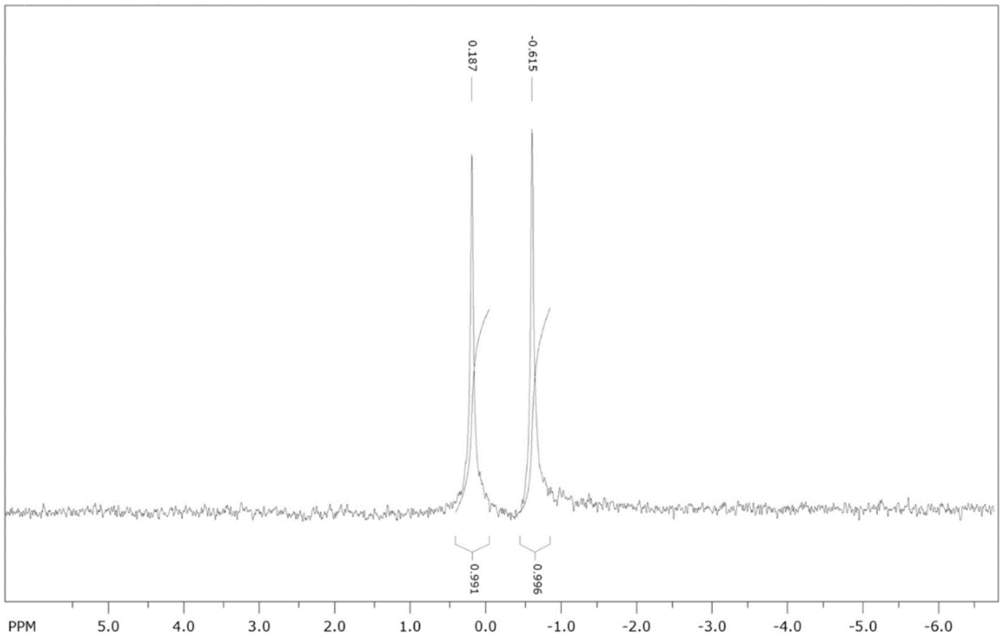

Figure S4. P NMR (121.5 MHz, D_2O) of compound 1a.

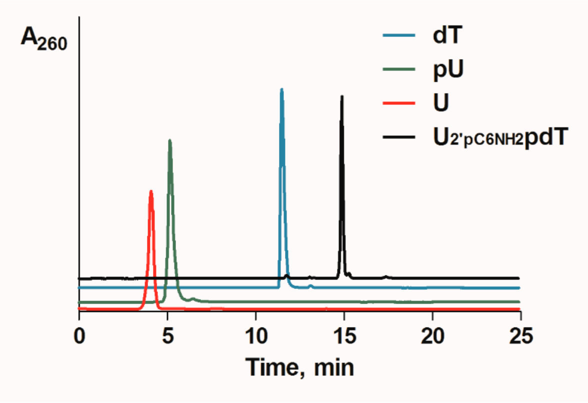

Figure S5. RP-HPLC profiles of purified dinucleotide 5'-$U_{2'pC6NH2}$pdT (**1a**) (black) and reference substances: thymidine (blue), uridine-5'-monophosphate (green), and uridine (red). The reverse phase-HPLC (RP-HPLC) analysis was performed on an Alphachrome high performance liquid chromatograph (EcoNova, Russia) with the use of a ProntoSil-120-5-C18 AQ (75 × 2.0 mm, 5.0 µm) column, applying a gradient elution of 0% (0–5 min), 0–7.5% (5–10 min), 7.5–12.5% (10–20 min), and 12.5–25% (20–25 min) of acetonitrile in 0.02 M triethylammonium acetate buffer, pH 7.0, at a flow rate 100 µL per min and detection at 260 nm.

1.5. Stability of 2′-Modified Dinucleotide 1a Containing Vicinal (2′- and 3′-) Phosphodiester Bonds

Chemical stability of the dinucleotide 5'-$U_{2'pC6NH2}$pdT (**1a**) was investigated under varying conditions (pH, temperature, and incubation time). Particular attention has been paid to the stability of vicinal phosphodiester bonds in the conditions that are supposed to be used in the further experiments with the 2'-modified oligoribonucleotides.

Table S1. Conditions chosen for **1a** stability test.

Condition	Buffer	Incubation
(A)	1mM HCl, pH 3.0	48 h at 25 °C
(B)	0.3M Na-acetate buffer, pH 5.2	48 h at 25 °C
(C)	0.3M Na-acetate buffer, pH 7.8	48 h at 25 °C
(D)	0.1M Na-cacodylate buffer, pH 7.4	10 min at 90 °C, then 48 h at 25 °C

The dinucleotide **1a** demonstrated high chemical stability at all of the chosen conditions (typical chromatograms of the products are shown in Figure S6). It was found that migration of vicinal phosphodiester bonds (at positions 3'- and 2'-) and the following cleavage of dinucleotide have not occurred. These observations are in agreement with previously published studies on the stability of 2′-branched oligonucleotides [2].

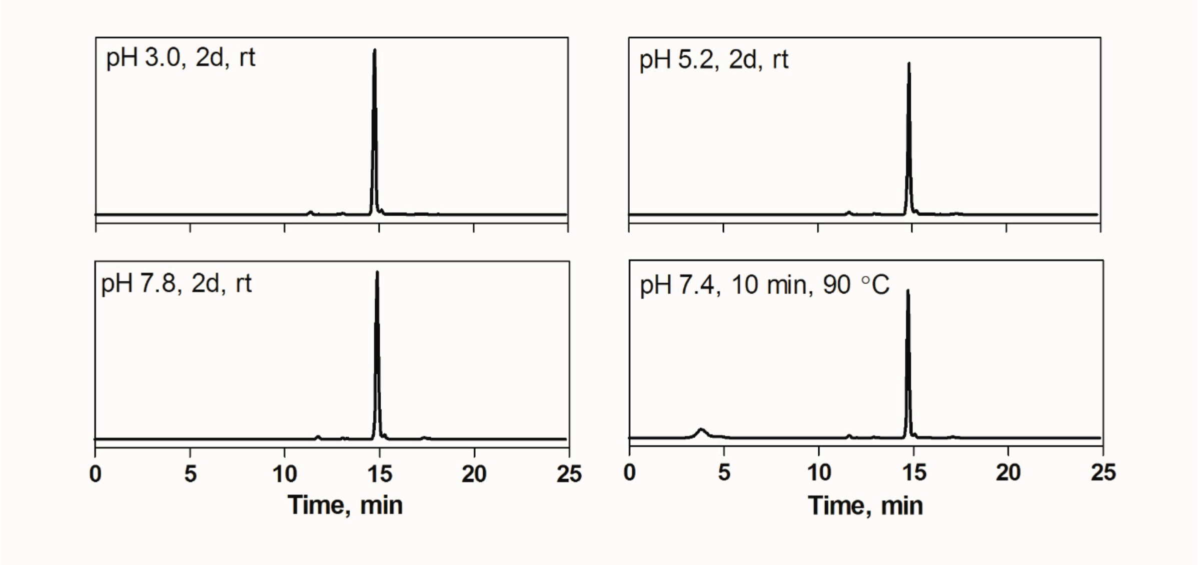

Figure S6. RP-HPLC profiles of dinucleotide **1a** after incubation under the selected conditions: See the caption of Figure S5 for details.

1.6. On-Column Post-Modification by Cholesteryl Chloroformate

Support-bound 2'-amino-C6-modified oligoribonucleotide (IV, see Scheme S2) was treated by cholesteryl chloroformate (4.5 mg and 10 μmol) and *N,N′*-diisopropylethylamine (2.5 μL, 2 mg, and approximately 15 μmol) in the mixture of anhydrous THF:CH_2Cl_2 (200 μL, 1:1 *v*/*v*) for 1 h at room temperature. Thereafter, the support-bound oligoribonucleotide was sequentially washed by THF, acetonitrile, and acetone and air-dried. Final deprotection and purification of the cholesterol-modified oligoribonucleotide from the crude mixture were performed as described in the Materials and Methods section.

1.7. Solid-Phase 2′ Modification by Formation of Carbamate Derivative

The support-bound 2'-*OH*/2'-*O*-TC oligoribonucleotide (III) was treated by the mixture of 1,1'-carbonyldiimidazole (5 mg and 30 μmol) and *N,N′*-diisopropylethylamine (5 μL, 4 mg, and approximately 30 μmol) in anhydrous THF (150 μL) for 1 h at room temperature (Scheme S3, step a). An excess of 1,1'-carbonyldiimidazole was removed from the reaction mixture, and the resulting support-bound oligoribonucleotide (V) was washed by anhydrous THF. After that, the 1,4-diaminobutane (2.6 mg and 30 μmol) in anhydrous THF (150 μL) was added, and the mixture was thoroughly mixed for 2 h at room temperature (Scheme S3, step b). Thereafter, support-bound oligoribonucleotide (VI) was sequentially washed by THF, acetonitrile, and acetone and air-dried. Final deprotection and cleavage of the 2'-modified oligoribonucleotide was performed as described in the Materials and Methods section. RP-HPLC profile of a crude amino-modified oligoribonucleotide **2f** (5'-r(ACG***U****ACGU)dT, ***U****-2'-*O*-(*N*-(4-aminobutyl)carbamoyl)uridine) showed a major peak of more than 80% of the integrated area (Figure S7). Following post-modification of the oligoribonucleotide**, 2f** was carried out with the use of cyanine3-NHS-ester, according to manufacturer-recommended protocol (http://www.lumiprobe.com), yielding major product **2g** (5'-r(ACG***U****ACGU)dT, ***U****—2'-cyanine3-labeled uridine) of high purity (>90%) (Figure S7, Table 1).

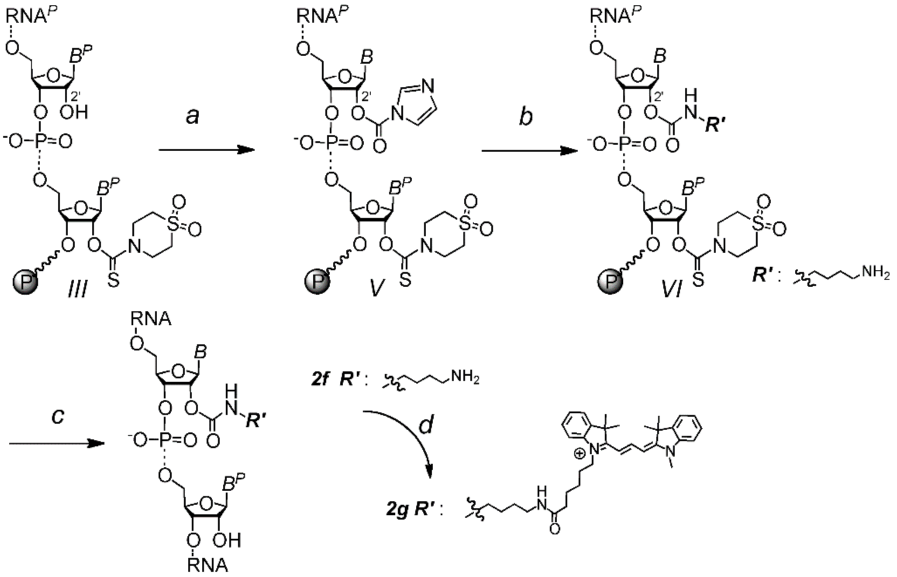

Scheme S3: Formation of 2'-carbamate. a) *N,N′*-carbonyldiimidazole, *N,N*-diisopropylethylamine (DIPEA), THF, 25 °C, 1 h; b) modifying amine (1,4-diaminobutane), DIPEA, THF, 25 °C, 2 h; c) $NH_2CH_2CH_2NH_2$ abs, 25 °C, 2 h; and d) cyanine3-NHS-ester, DMSO, 0.1 M $NaHCO_3$, pH 8.3. B^P(B)—(un)protected nucleobase. RNA^P(RNA)—(un)protected oligoribonucleotide.

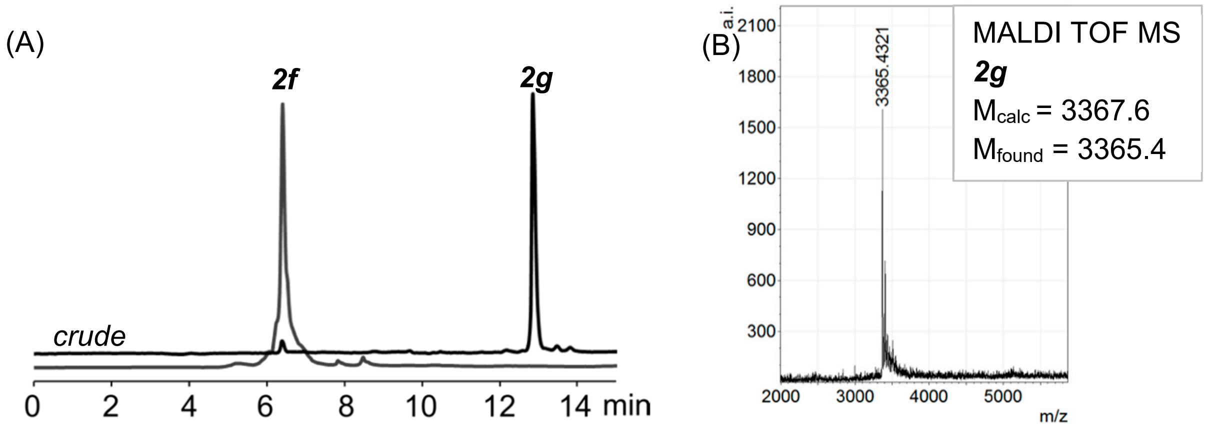

Figure S7. (**A**) RP-HPLC profiles of crude oligonucleotides **2f** and **2g**; (**B**) MALDI-TOF spectra of cyanine3-labeled oligoribonucleotide **2g**.

1.8. Post-Modification in Solution

For 2'-cyanine7-modified oligoribonucleotide **2*c*,** a solution of cyanine7-NHS-ester (2 mg, 3 μmol) in DMSO (130 μL) was added to a solution of **2a** (≈ 0.1 μmol) in 0.1 M $NaHCO_3$, pH 8.3 (40 μL), and the reaction was stirred at rt overnight. The crude mixture was precipitated by ten volumes of $NaClO_4$ (2%) in acetone and washed by acetone. The oligoribonucleotide **2*c*** was purified by denaturing PAGE (15%).

For 2'-pyrene-modified oligoribonucleotide **2e,** a mixture of a solution of **2e** (≈ 0.1 μmol) in 0.2 M triethylammonium acetate buffer, pH 7.0 (50 μL); DMSO (45 μL); pyrene azide 1 in DMSO (10 mM and 3 μL); and ascorbic acid in H_2O (to final concentration of 0.5 mM) was degased by bubbling Ar. After that, a solution of 10 mM copper (II)-TBTA complex in 55% aq DMSO (5 μL) was added to the mixture; then, the reaction mixture was degased and then stirred at rt overnight. The crude mixture was precipitated and purified as described above.

1.9. Synthesis of 2′-Cholesterol-Containing Conjugates of Sense Strand of siRNA

Series of 2'-functionalized 21-mer oligonucleotides **8a–b–10a–b** (Table S2) was synthesized at a 0.2 µmol scale. The acetylation of 5'-hydroxyl has been replaced with modification by a 5'-alkyne-modifier to introduce fluorescent dye (Fluorescein) by CuAAC chemistry in solution as one of *post*-modification steps. After that, subsequent steps of selective deprotection and *on-column* 2'-modification of the 2'-*OH*/2'-*O*-TC RNA by 5'-amino-modifier C6 were performed as described above. To enable monitoring of the efficiency of the 2'-modification by 5'-amino-modifier *C*6 phosphoramidite, oligonucleotides **8a–10a** were deprotected and released from solid support by neat ethylenediamine as described in the Materials and Methods section. Analysis of the products by denaturing PAGE (15%, 19:1) showed that the conversion was almost complete (>90%) (Figure S9). 2'-cholesterol-functionalized oligonucleotides **8b–10b** were prepared based on support-bound **8a–10a** as described in Section 1.6. with the following final deprotection and labeling by CuAAC chemistry in solution (similar to the procedure described in Section 1.8.). The conversion after both reactions was approximately 45–50% (PAGE (15%, 19:1), Figure S9). The oligoribonucleotides **8a-b–10a-b** were purified by denaturing PAGE (15%, 30:0.5).

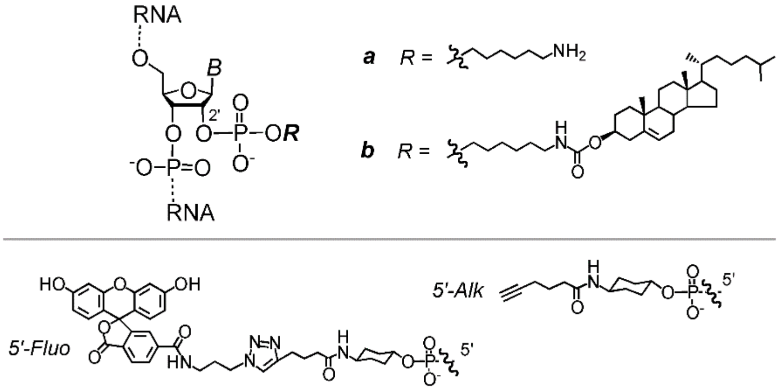

Figure S8. Structures of modifications introduced in **8a-b–10a-b**.

Table S2. Conjugates of sense strand siRNA used in this study [a]

No.	Sequence[b]	R Group
**8a**	5′-*Alk*-r(GCCACAACGUCUAUAUCA***U****)dTdT	**a**
**8b**	5'-*Fluo*-r(GCCACAACGUCUAUAUCA***U****)dTdT	**b**
**9a**	5′-*Alk*-r(GCCACAACGUCUAU***A****UCAU)dTdT	**a**
**9b**	5'-*Fluo*-r(GCCACAACGUCUAU***A****UCAU)dTdT	**b**
**10a**	5′-*Alk*-r(GCCACAACGUC***U****AUAUCAU)dTdT	**a**
**10b**	5'-*Fluo*-r(GCCACAACGUC***U****AUAUCAU)dTdT	**b**

[a] 5'-r(NN…N)—oligoribonucleotide, dT—3'-terminal thymidine, ***N****—2'-modified nucleoside, Alk—alkyne containing group, Fluo—fluorescein (6-isomer).

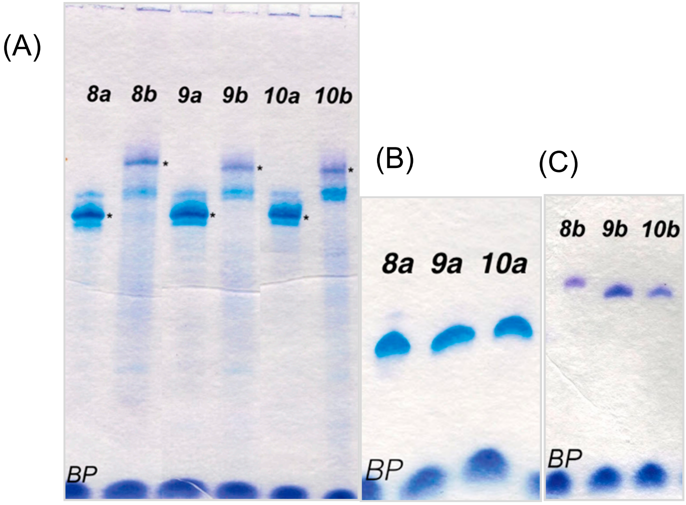

Figure S9. PAGE profiles of crude (**A**) and purified (**B**, **C**) oligonucleotides **8a**–**b**–**10a**–**b**: migration of BP (bromophenol blue) corresponds to 3-6 nt.

1.10. Mass-Spectrometry Analysis of the 2′ Conjugates of the Sense Strand of siRNA

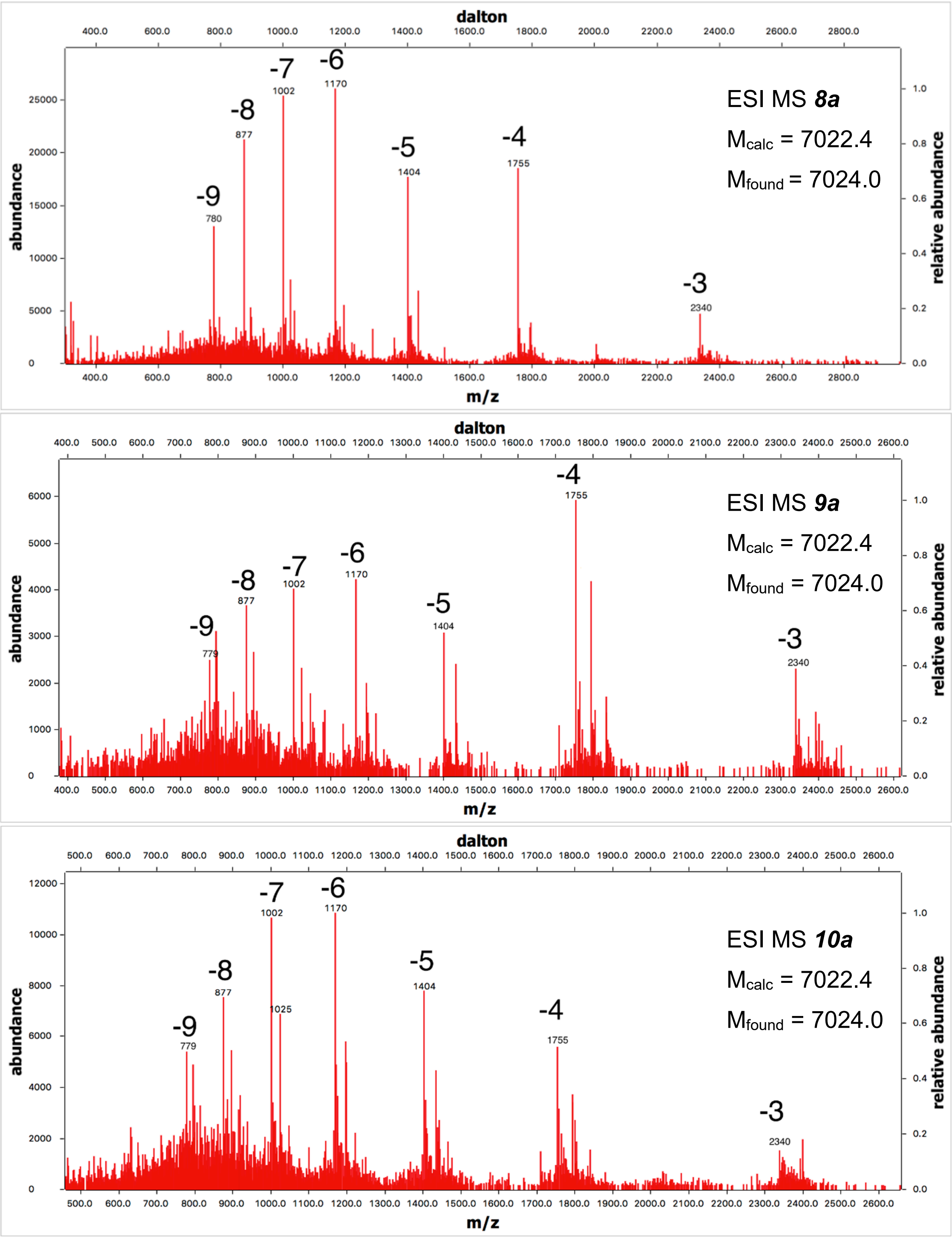

Figure S10. Mass spectrometry profiles of the obtained 2′-functionalized RNAs on an example of 2′-cholesterol conjugates of a sense strand of siRNA.

1.11. HPLC Analysis of the 2′-Functionalized Oligoribonucleotides

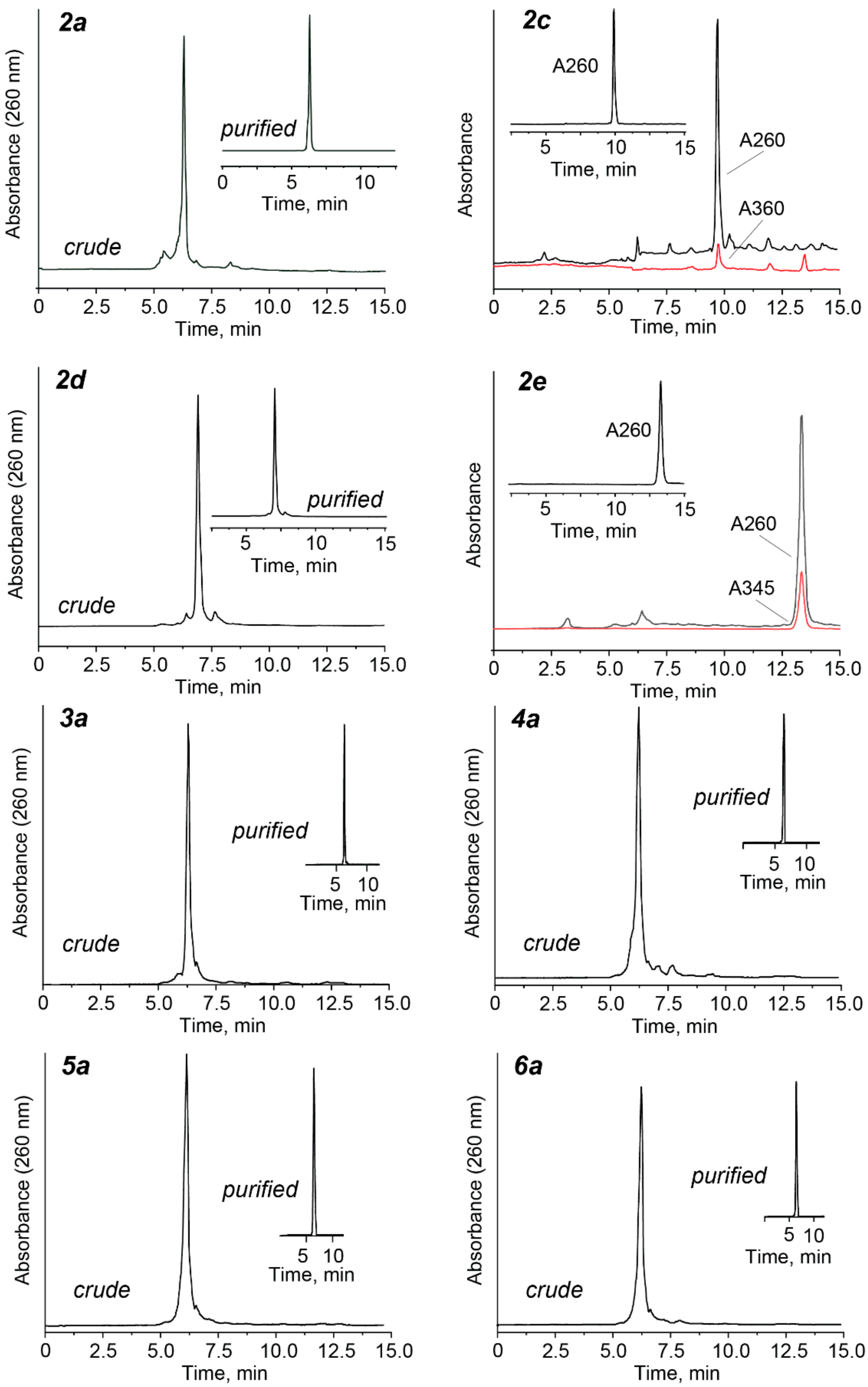

Figure S11. Reverse phase HPLC profiles of the 2′-amino- **2a**, 2′-Cy7- **2c**, 2′-alkyne- **2d** and 2′-pyrene-modified oligoribonucleotide **2e** and 2′-amino-modified oligoribonucleotides **3a**–**6a**.

2. Physicochemical Studies

2.1. UV-Visible Absorption Spectra

UV-visible absorption spectra of purified 2'-modified oligoribonucleotides were recorded on an Eppendorf BioSpectrometer in a buffer containing 0.1 M NaCl, 10 mM sodium cacodylate, pH 7.4, and 1 mM Na_2EDTA.

The absorption ratios at 260 and 345 nm in the spectra of the 2'-pyrene-modified probe ***2e*** complied with the calculated ratio of the oligonucleotide and pyrene parts with consideration of their molar absorption coefficients. The molar absorption coefficients of the 2'-pyrene-modified oligoribonucleotides were calculated as a sum of the molar absorption coefficients of the corresponding oligonucleotides and one residue of pyrene attached to the oligomer (24000 M^{-1} cm^{-1} at 260 nm) [3].

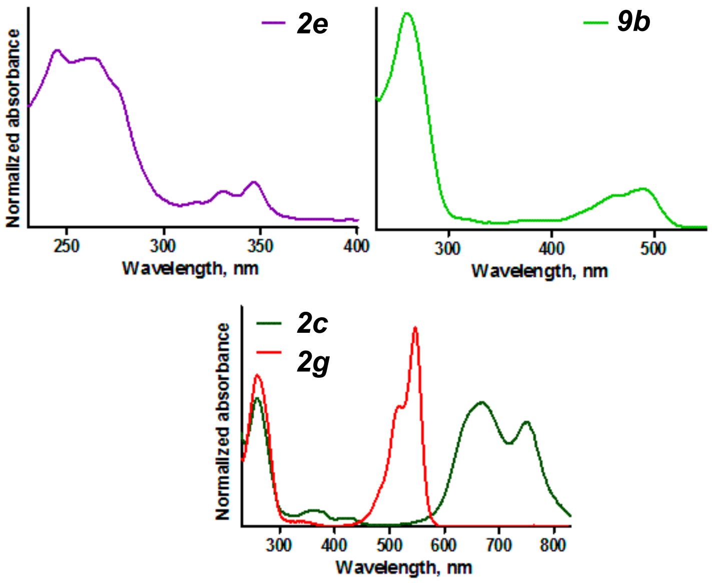

Figure S12. UV/vis-spectra of purified dye-labeled 2′-conjugates of RNA.

2.2. Thermal Denaturation Dtudies

Thermal denaturation experiments were performed on a Cary 300 BioMelt Spectrophotometer equipped with Temperature Probes Accessory Series II (Varian Inc., Australia) in medium salt buffer containing 0.1 M NaCl, 10 mM Na cacodylate, pH 7.4, and 1 mM Na_2EDTA. The oligonucleotides (1.0 µM each strand) were mixed, denatured by heating up to 92 °C, and subsequently cooled to the starting temperature of experiment. The rate of temperature change was 0.5 °C/min. The thermal denaturation curves of duplexes of 2'-*O*-(6-aminohexyl)phosphate-functionalized oligoribonucleotides with their RNA targets display sigmoidal monophasic transitions (Figure S13) similarly to the reference duplexes (data not shown). Duplex melting temperatures (T_m (ΔT_m), °C) (see Table 2) were calculated using the thermodynamic parameters ΔG^{o}_{298}, ΔH°, and ΔS° (see Table S3) that have been obtained by fitting procedure of UV-melting curves registered at two different wavelengths (260 and 270 nm) [4] according to two state model [5]. The calculated parameter determination error did not exceed 5–10%.

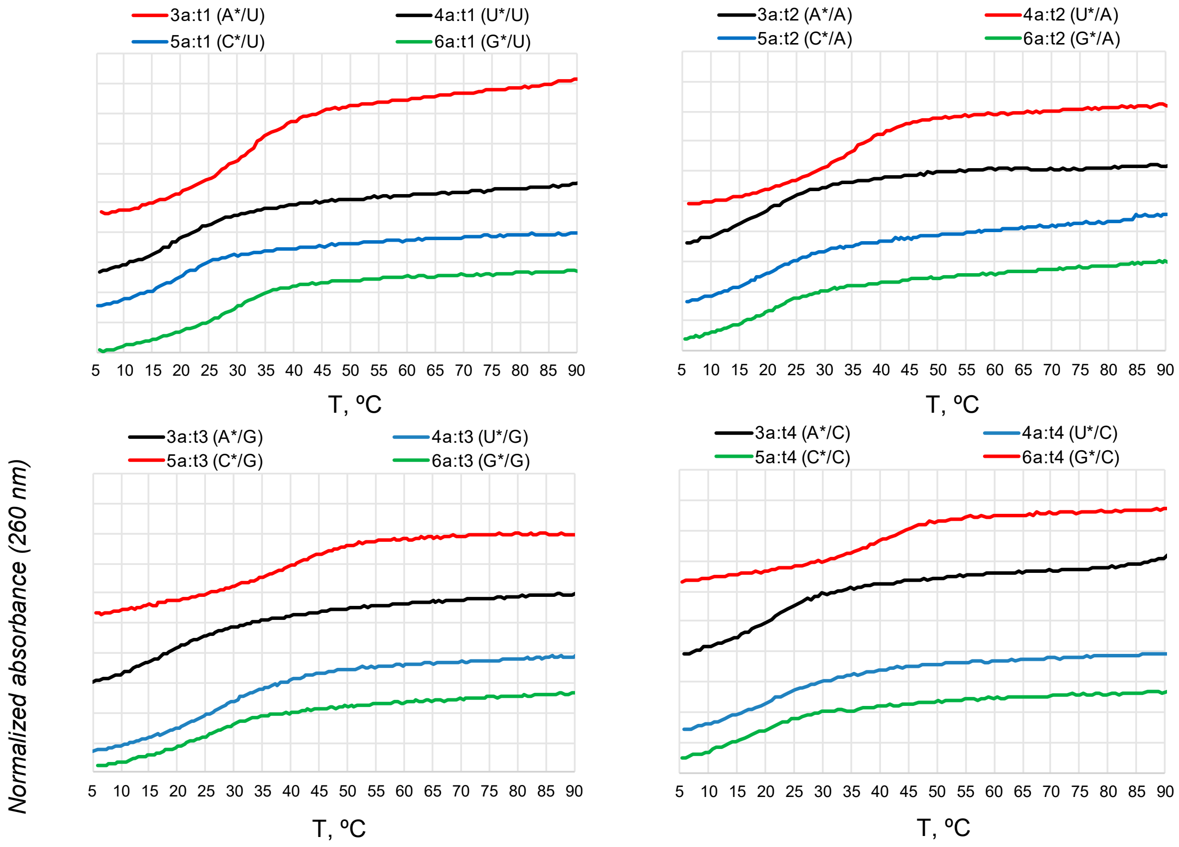

Figure 13. Thermal denaturation curves obtained from heating experiment monitored at 260 nm in 0.1 M NaCl, 10 mM sodium cacodylate, pH 7.4, and 1 mM Na_2EDTA with 1.0 μM each strand of oligonucleotide: See sequences of the 2'-*O*-(6-aminohexyl)phosphate-functionalized oligoribonucleotides **3a**–**6a** and their corresponding targets **t1**–**t4** in Table 2.

Table S3. Thermodynamic parameters for formation of duplexes between **3–6**, **3a–6a** and complementary or centrally mismatched RNA targets.

ON	Sequence [a]	Targets: t1, 3'-r(CACUUUACG)			t2, 3'-r(CACUAUACG)			t3, 3'-r(CACUGUACG)			t4, 3'-r(CACUCUACG)		
		ΔG^{o}_{298} ($\Delta\Delta G^{o}_{298}$), kJ·mol^{-1}	$\Delta H°$ ($\Delta\Delta H°$), kJ·mol^{-1}	$-T_{298}\Delta S°$ ($\Delta(T_{298}\Delta S°)$), kJ·mol^{-1}	ΔG^{o}_{298} ($\Delta\Delta G^{o}_{298}$), kJ·mol^{-1}	$\Delta H°$ ($\Delta\Delta H°$), kJ·mol^{-1}	$-T_{298}\Delta S°$ ($\Delta(T_{298}\Delta S°)$), kJ·mol^{-1}	ΔG^{o}_{298} ($\Delta\Delta G^{o}_{298}$), kJ·mol^{-1}	$\Delta H°$ ($\Delta\Delta H°$), kJ·mol^{-1}	$-T_{298}\Delta S°$ ($\Delta(T_{298}\Delta S°)$), kJ·mol^{-1}	ΔG^{o}_{298} ($\Delta\Delta G^{o}_{298}$), kJ·mol^{-1}	$\Delta H°$ ($\Delta\Delta H°$), kJ·mol^{-1}	$-T_{298}\Delta S°$ ($\Delta(T_{298}\Delta S°)$), kJ·mol^{-1}
**3**	5'-r(GUGAAAUG)dC	−33	−314	270	−20 (+17)	−206 (+110)	178 (−90)	−21 (+20)	−154 (+121)	127 (−98)	−23 (+16)	−219 (+28)	189 (−10)
**4**	5'-r(GUGAUAUG)dC	−25 (+8)	−188 (+126)	157 (−113)	−37	−316	268	−31 (+10)	−187 (+88)	150 (−75)	−22 (+17)	−196 (+51)	167 (−32)
**5**	5'-r(GUGACAUG)dC	−25 (+8)	−106 (+208)	78 (−192)	−23 (+14)	−188 (+128)	158 (−110)	−41	−275	225	−31 (+8)	−226 (+21)	188 (−11)
**6**	5'-r(GUGAGAUG)dC	−32 (+1)	−291 (+23)	250 (−20)	−23 (+14)	−197 (+119)	168 (−100)	−24 (+17)	−158 (+117)	128 (−97)	−39	−247	199
**3a**	5'-r(GUGA**A***AUG)dC	−27	−210	176	−18 (+11)	−192 (+30)	166 (−19)	−22 (+13)	−129 (+91)	103 (−75)	−20 (+15)	−180 (+93)	154 (−75)
**4a**	5'-r(GUGA**U***AUG)dC	−19 (+8)	−171 (+39)	145 (−31)	−29	−222	185	−27 (+8)	−141 (+79)	110 (−68)	−22 (+13)	−144 (+129)	117 (−112)
**5a**	5'-r(GUGA**C***AUG)dC	−18 (+9)	−204 (+6)	180 (+4)	−20 (+9)	−170 (+52)	144 (−41)	−35	−220	178	−18 (+17)	−193 (+80)	168 (−61)
**6a**	5'-r(GUGA**G***AUG)dC	−25 (+2)	−237 (−27)	203 (+27)	−20 (+9)	−181 (+41)	155 (−30)	−23 (+12)	−183 (+37)	153 (−25)	−35	−273	229

[a] 5'-r(NN…N)—oligoribonucleotides, dC—3'-terminal deoxycytidine, **N***—2'-O-(6-aminohexyl)phosphate nucleotide monomer (Table 1). $\Delta\Delta G^{298}$, $\Delta\Delta H$, and $\Delta(T^{298}\Delta S)$ are calculated relative to fully matched duplexes. Thermal denaturation buffer: 0.1 M NaCl, 10 mM sodium cacodylate, pH 7.4, and 1 mM Na_2EDTA; the concentration of each strand was 1 μM.

2.3.CD Spectra of Duplexes of the 2′-Functionalized Oligoribonucleotides

CD spectra were recorded on a Jasco J-600 spectropolarimeter (JASCO Co., Ltd., Japan) connected to LKB 2219 MULTITERM II water bath (LKB, Sweden) at 15 μM concentration of oligoribonucleotide duplexes in cacodylate buffer (0.1 M NaCl, 10 mM sodium cacodylate, pH 7.4, and 1 mM Na_2EDTA) at 10 °C. Spectra were recorded using a 0.1 cm path length quartz cell at 50 nm·min^{-1} (accumulation: 5) with a response time of 0.5 s, a bandwidth of 2.0 nm, and a resolution of 1 nm. A buffer baseline was subtracted from each spectrum, which was recorded in a range of 330–200 nm.

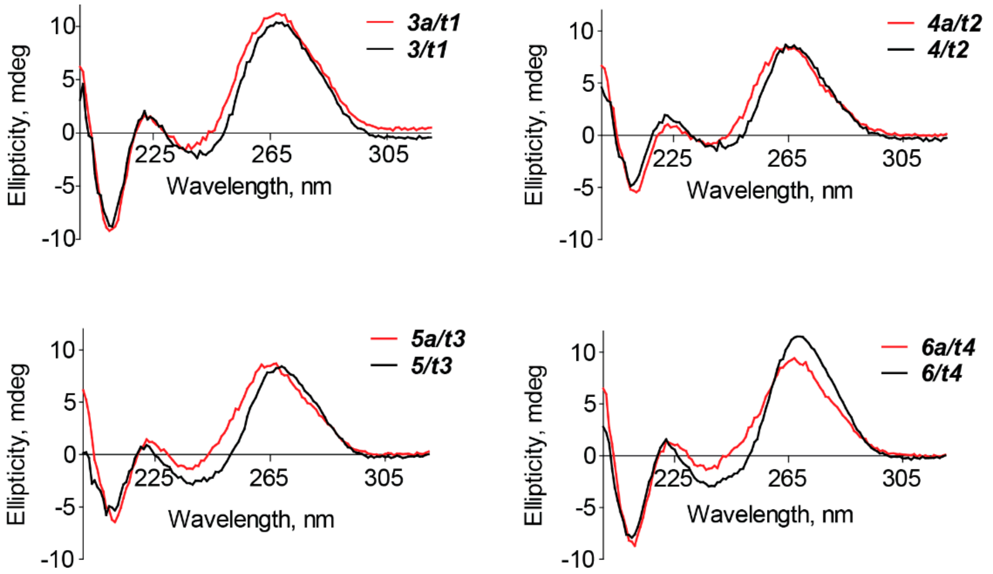

Figure S14. Overlay of CD spectra of duplexes of the 2'-*O*-(6-aminohexyl)phosphate-functionalized oligoribonucleotides with their corresponding targets (**3a/t1**–**6a/t4**) and reference unmodified duplexes (**3/t1**–**6/t4**), C_{RNA} = 15 μM.

3. Biological Evaluation of 2'-Cholesterol-Containing siRNA Conjugates

3.1. Cell Culture

The HEK293 Phoenix human embryonic kidney cell line was kindly provided by Prof. O.L. Serov (Institute of Cytology and Genetics, Novosibirsk, Russia). Cells were cultured in Dulbecco's modified Eagle medium (DMEM) supplemented with 10% fetal bovine serum, 1X NEAA (Gibco, Thermo Fisher Scientific, Waltham, MA, USA), L-glutamine, and 100 units of penicillin/sreptomycin (Gibco, Thermo Fisher Scientific, Waltham, MA, USA). Cells were cultured at 37 °C with a mixture of air and 5% CO_2.

3.2. siRNAs

The lyophilized RNA strands (see Table S3) were dissolved in a buffer containing 137 mM NaCl, 2.7 mM KCl, and 10 mM phosphate buffer, pH 7.4; the final concentration of siRNA was 20 μM. The solution was heated at 90 °C for 2 min and then slowly cooled to rt for 1 h.

3.3. Cellular Uptake of the 2′-Cholesterol-Conjugated siRNA in the HEK293 Phoenix Cell Line

3.3.1. Transfection

HEK293 Phoenix cells were plated on 48-well plates or on 8-well μ-slides (ibidi) 24 h before transfection to achieve 70–90% confluence at transfection. Before transfection, the culture medium was changed to the Opti-MEM medium (Gibco). For lipofectamine-mediated transfections, we used 5 pmole of fluorescein-labeled siRNA and 0.5 μL lipofectamine 3000 without P3000 reagent (Thermo

Fisher Scientific, USA) per well (final concentration of siRNAs ~ 0.027 uM). The transfection was performed according to manufacturer instructions. For siRNA free uptake, we diluted siRNAs in OptiMem medium to a final concentration of 1 uM and applied it to cells. The cells were incubated for 4 h at 37 °C with a mixture of air and 5% CO_2.

3.3.2. Flow Cytometry

Cells were washed with PBS, detached using 0.25% trypsin/EDTA solution, pelleted by centrifugation, and resuspended in PBS. Flow cytometry was performed using BD FACS Area I. Control (GFP-negative and GFP-positive) cells were used to setup gates.

3.3.3. Confocal Microscopy

HEK293 Phoenix cells were transfected on 8-well μ-slides (ibidi) as described above. After the siRNA transfection into cells, they were fixed with 4% paraformaldehyde in 1 × PBS for 10 min at rt and washed twice with 1 × PBS. Fluorescence imaging experiments were carried out using a LSM 780 NLO Zeiss laser scanning confocal microscope based on AxioObserver Z1 with diode (405 nm) and argon (514 nm) lasers and Plan APOCHROMAT 20 ×/0.8, EC Plan NEOFLUAR 40 ×/1.3, and Plan APOCHROMAT 63 ×/1.4 Oil DIC objective lens and equipped with an AxioCam MRm digital camera (Zeiss, Germany). Fluorescence visualization of fluorescein-labeled siRNAs in cells was performed at rt using an excitation wavelength of 488 nm (Fluorescein). Images were obtained and analyzed with the use of ZEN 2012 (Carl Zeiss Microscopy GmbH, Germany) and ImageJ software. All images were recorded in gray scale and converted into green and cyan pseudo-color using the software.

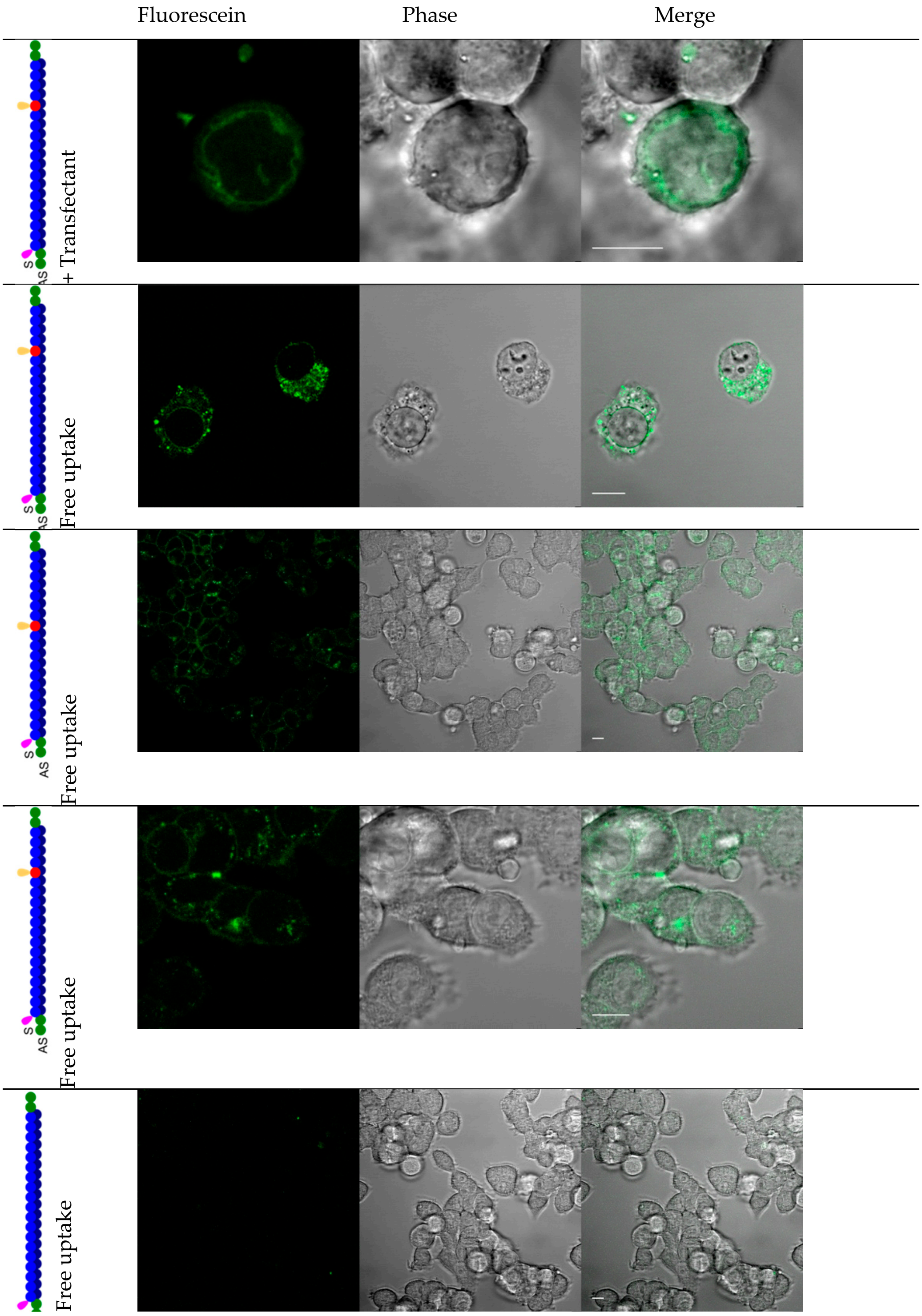

Figure S15. Selected fluorescence microscopy images of HEK293 Phoenix cells treated with synthesized siRNAs in the absence (1 uM, free uptake) and in the presence (~ 0.027 uM of siRNA) of transfection agent lipofectamine 3000 for 4 h at 37 °C in OptiMEM medium. The scale bar is 10 μm.

References

1. M.J. Damha, R. T. Pon, K. K. Ogilvie, *Tetrahedron Lett.* **1985**, *26*, 4839–4842.
2. H. Tsuruoka, K. Shohda Ki, T. Wada, M. Sekine, *J. Org. Chem.* **1997**, *62*, 2813–2822.
3. M. I. Dobrikov, S. A. Gaidamakov, A. A. Koshkin, T. I. Guinutdinov, N. P. Luk'ianchuk, G. V Shishkin, V. V Vlasov, Russ. *J. Bioorg. Chem.* **1997**, *23*, 171–178.
4. D. V Pyshnyi, A. A. Lomzov, I. A. Pyshnaya, E. M. Ivanova, *J. Biomol. Struct. Dyn.* **2006**, *23*, 567–580.
5. M. Petersheim, D. H. Turner, *Biochemistry* **1983**, *22*, 256–263.

© 2020 by the authors. Licensee MDPI, Basel, Switzerland. This article is an open access article distributed under the terms and conditions of the Creative Commons Attribution (CC BY) license (http://creativecommons.org/licenses/by/4.0/).
